# Optogenetic dissection of RET signaling reveals robust activation of ERK and enhanced filopodia-like protrusions of regenerating axons

**DOI:** 10.1186/s13041-023-01046-6

**Published:** 2023-07-04

**Authors:** Bobae Hyeon, Heeyoung Lee, Nury Kim, Won Do Heo

**Affiliations:** 1 Department of Life Sciences, Korea Advanced Institute of Science and Technology (KAIST), 291 Daehak-Ro, Yuseong-Gu, Daejeon, 305-701 Republic of Korea; 2 Center for Cognition and Sociality, Institute for Basic Science (IBS), Daejeon, Republic of Korea; 3 Korea Advanced Institute of Science and Technology (KAIST), KAIST Institute for the BioCentury, 291 Daehak-Ro, Yuseong-Gu, Daejeon, 305-701 Republic of Korea

**Keywords:** Optogenetics, RET, GDNF, PHR, Neuron, Signaling, Actin, AKT, MAPK, ERK, TrkB, Filopodia, Cdc42, Regeneration, Dopaminergic neuron

## Abstract

RET (REarranged during Transfection) is a receptor tyrosine kinase that transduces various external stimuli into biological functions, such as survival and differentiation, in neurons. In the current study, we developed an optogenetic tool for modulating RET signaling, termed optoRET, combining the cytosolic region of human RET with a blue-light–inducible homo-oligomerizing protein. By varying the duration of photoactivation, we were able to dynamically modulate RET signaling. Activation of optoRET recruited Grb2 (growth factor receptor-bound protein 2) and stimulated AKT and ERK (extracellular signal-regulated kinase) in cultured neurons, evoking robust and efficient ERK activation. By locally activating the distal part of the neuron, we were able to retrogradely transduce the AKT and ERK signal to the soma and trigger formation of filopodia-like F-actin structures at stimulated regions through Cdc42 (cell division control 42) activation. Importantly, we successfully modulated RET signaling in dopaminergic neurons of the substantia nigra in the mouse brain. Collectively, optoRET has the potential to be developed as a future therapeutic intervention, modulating RET downstream signaling with light.

**Supplementary Information:**

The online version contains supplementary material available at 10.1186/s13041-023-01046-6.

## Introduction

RET (REarranged during Transfection), encoded by the *c-ret* proto-oncogene, is a transmembrane receptor belonging to the receptor tyrosine kinase (RTK) family [1–5]. It transduces various external stimuli into biological responses, such as survival and differentiation, in a wide range of neuronal populations, including dopaminergic (DA) neurons, spinal motor neurons and enteric neurons, as well as in non-neuronal cell populations [1–4]. Dysregulation of RET signaling has been reported in many pathologies, including developmental disorders and cancers in humans [2, 6].

The structure of RET can be subdivided into an extracellular region (EXR), a transmembrane domain (TMD), and a cytoplasmic region (CYR; Additional file 1: Fig. S1). The RET-EXR is composed of four cadherin-like domains (CLDs 1–4) and a cysteine-rich region (CRD), both of which require the binding of calcium for their proper folding in the extracellular space [7]. The RET-CYR is composed of the juxtamembrane domain (JMD), tyrosine kinase domain (TKD) and the tail; its sequence after the splicing site, amino acid (aa) 1063, varies according to the nomenclature of human RET, as reflected in the three splicing variants of RET (RET9, RET43 and RET51), named according to the number of additional amino acids after the splicing site [6, 8].

RET activation requires assembly of RET with its ligand and a ligand-binding co-receptor into a dimer or oligomer of a tripartite complex [9–11]. Five different RET ligands and their respective co-receptors have been identified. These include the glial cell line-derived neurotrophic factor (GDNF) family ligands (GFLs) GDNF, neurturin (NRTN), artemin (ARTN) and persephin (PSPN), and their respective preferred co-receptors, known as GFL receptor α (GFRα) 1–4; also included are growth and differentiation factor 15 (GDF15) and its co-receptor, GFRα-like protein (GFRAL) [2, 11–13]. In general, RET co-receptors are glycosylphosphatidylinositol (GPI)-anchored proteins located in lipid rafts—microdomains at the plasma membrane (PM)—and their binding to dimeric ligands recruits RET into the lipid raft for activation within a cell [6] (Additional file 1: Fig. S1). However, there are soluble forms of GFRα (sGFRα), produced by cells such as glial cells, that can mediate the activation of RET outside lipid rafts, complexing with the sGFRα and a RET molecule in another cell [14].

The formation of a tripartite complex brings at least a pair of RET receptors into sufficient proximity to trans-autophosphorylate ten tyrosine residues within the common region of the three RET-CYRs [6]. Several of these phosphorylated tyrosine residues (pY), in turn, serve as docking sites for various adaptor or effector proteins containing Src homology 2 (SH2) or phosphotyrosine-binding (PTB) domains (Additional file 1: Fig. S1) [2, 6]. Among these sites, pY905 in the activation loop is required for the full kinase activity of RET while also serving as the docking site for growth factor receptor-bound proteins 7 and 10 (Grb7/10) [8]. pY981 is the docking site for Src and SH2-Bβ (SH2 adaptor protein Bβ), which mediate survival and neurite outgrowth, respectively [15–17], and there is growing evidence that it is also necessary for full activation of RET and consequent transduction of downstream signaling [18, 19]. pY1015 is the docking site for phospholipase Cγ (PLCγ) and subsequent activation of protein kinase C (PKC) and Ca^2+^-signaling cascades [6]. pY1062 is a critical residue that acts as a signaling hub, complexing with multiple adaptor proteins including fibroblast growth factor receptor substrate 2 (FRS2), Src-homology collagen (Shc), insulin receptor substrate 1 and 2 (IRS1/2) and downstream of kinase (DOK)-4, -5 and -6, to activate phosphatidylinositol-3 kinase/protein kinase B (PI3K/AKT) and Rat sarcoma virus/mitogen-activated protein kinase (Ras/MAPK) pathways [2, 18, 20, 21]. FRS2 recruits Grb2, activating Ras/MAPK pathways, and complexes with the SH2-containing protein, tyrosine phosphatase-2 (SHP-2) to cooperatively bind to pY687 in the RET-JMD, which is essential for the sustained activation of MAPK [21]. Shc recruits Grb2 for Ras/MAPK pathways but additionally complexes with Grb2-associated binding protein 1 and 2 (GAB1/2) for PI3K/AKT pathways [21]. Interestingly, recruitment of these four adaptor proteins is mediated by their PTB domains, which compete for binding to the amino acid sequence, NKL-pY1062 of RET [2, 21, 22]. The mechanisms that regulate the binding of these proteins are likely to determine biological outcomes. For instance, FRS2 and Shc are recruited to plasma membrane non-raft and lipid raft domains, respectively [21]. In general, active RET first associates with FRS2 in the lipid raft and then translocates outside the raft to associate with Shc in a sequential manner [2, 21].

Notably, alternative signaling receptors for RET ligands have also been identified and characterized [2]. For example, DA neurons in the midbrain, where RET is highly expressed, have been reported to also express other GDNF receptors, including neuronal cell adhesion molecule (NCAM), integrins, syndecan-3, and N-cadherin [23]. The existence of these RET-independent signaling pathways with a deep pool of RET ligands not only adds a layer of sophistication that complicates our understanding of RET signaling, but it also makes it more difficult to develop therapeutic strategies for treating diseases related to RET signaling.

Over the last decade, we and others have developed an optogenetic toolbox for modulating various RTKs, including tropomyosin receptor kinases (TRKs), fibroblast growth factor receptors (FGFRs) and, epidermal growth factor receptor (EGFR), among others [24–26]. Because of their light-activation property, these optogenetic RTK-modulating tools, designated optoRTKs, have been demonstrated to induce receptor-specific biological functions with a high spatiotemporal resolution in response to stimulation with the appropriate wavelength of light. Prominent examples include the directed migration of fibroblast cells and determination of axonal fate in cultured neurons through activation of optoFGFR1 and optoTrkB, respectively [25, 27]. An optogenetic RET, designated Opto-hRET, was also developed by the Janovjak group, utilizing the blue-light–inducible dimerization property of the light oxygen voltage-sensing (LOV) domain [26]. However, the functionality of the tool was only characterized in non-neuronal cell lines [26]. More recently, this group reported pioneering work on the application of Opto-hRET to treat Parkinson’s disease (PD) [28], although this work was performed in a *Drosophila* model, which has a DA system that is not directly comparable to that of mammalian systems.

In the current study, we developed another optogenetic tool for the modulation of RET signaling, termed optoRET, utilizing only the common RET-CYR of the three RET variants in humans and taking advantage of the blue-light–inducible homo-oligomerization property of the photosensitive domain (PHR) from *Arabidopsis* cryptochrome 2 (CRY2) [29, 30]. To demonstrate the versatile and dynamic control of RET downstream signaling by light, we applied this tool in cultured neurons and DA neurons of the substantia nigra (SNc) in mice.

## Methods

### Plasmid construction

For generation of the pcDNA3.1-CMV-RET^aa1−1072^-PHR-mCitrine construct, termed optoRET^full−length^, human RET9 (aa 1–1072) was amplified by polymerase chain reaction (PCR) from human brain total RNA (Clontech) using the primer pair, 5′-CGT CAG ATC CGC TAG CCA CCA TGG CGA AGG CGA CGT CCG GT-3′ (forward) and 5′ GGC GAC CGG TGG ATC CCC GAA TCT AGT AAA TGC ATG GGA AAT-3′ (reverse), and inserted into the pcDNA3.1-CMV-PHR-mCitrine construct [24] using the In-Fusion cloning system (Clontech), according to the manufacturer’s instructions. For generation of the pcDNA3.1-CMV-Lyn-RET^aa658−1062^-PHR-mCitrine construct, the common RET-CYR of the three RET variants (aa 658–1062) was PCR-amplified with the primers, 5′-ATT CCT CGA GGg atc atc ACT GCT ACC ACA AG-3′ (forward) and 5′ CAT ACC TGT cgg atc ATA GAG TTT GTT TTC A-3′ (reverse) and inserted into the pcDNA3.1-CMV-Lyn-cytTrkB-PHR-mCitrine construct [27] using the In-Fusion cloning system. To minimize basal activity, we performed site-directed mutagenesis to mutate residue E281 to alanine (PHR^E281A^) [30, 31]. This final form of the construct—pcDNA3.1-CMV-Lyn-RET^aa658−1062^-PHR^E281A^-mCitrine—was termed optoRET. An additional D387A mutation in the PHR region of optoRET [24, 32], introduced by PCR, created a light-insensitive form of optoRET construct (pcDNA3.1-CMV-Lyn-RET^aa658−1062^-PHR ^E281A,D387A^-mCitrine) for use as a control. For in vitro analyses, we utilized modified versions of previously generated biosensors in which the respective fluorescent proteins of the original forms were replaced, as follows: pcDNA3.1-CMV-hGrb2-GFP, pcDNA3.1-CMV-mCherry-AKT-PH [25, 33], pcDNA3.1-CMV-mCherry-LifeAct [25, 34] and pcDNA3.1-CMV-ERK-KTR-FusionRed [35].

Adeno-associated viruses (AAVs), in the form of pAAV2-ITR-transgene vectors, were generated as follows. pAAV-CamKIIα(0.4)-DIO-optoRET(HA) and pAAV-hSyn1-DIO-optoRET(HA) were constructed from the respective constructs, pAAV-CamKIIα(0.4)-DIO-Opto-cytTrkB(E281A)-HA (Addgene #180588) and pAAV-hSyn1-DIO-Opto-cytTrkB(E281A)-HA (Addgene #180590), by replacing Lyn-cyTrkB-PHR sequences with the Lyn-RET^aa658−1062^-PHR^E281A^ sequences of optoRET using the *Nhe*I/*Age*I restriction enzyme sites [31]. pAAV-CamKIIa(0.4)-ERK-KTR-Clover and pAAV-CamKIIa(0.4)-mScarlet-IRES2-CRE were constructed from pAAV-CamKIIa(0.4)-EGFP (Addgene #50469) by replacing the EGFP sequences with the ERK-KTR-Clover sequences (Addgene #59150) or with mScarlet (Addgene #85042), IRES2 and CRE (Addgene #51268 and #51267), respectively.

### AAV production

AAVs were produced using a triple transfection system employing polyethyleneimine (PEI). Human embryonic kidney (HEK) 293T cells, used as packaging cells, were cultured in Dulbecco’s modified Eagle’s medium (DMEM; Gibco, cat. #11965092), supplemented with 10% fetal bovine serum (FBS) at 37 °C in a humidified 10% CO_2_ environment. A DNA mixture consisting of a transgene vector, a packaging vector (pRC-DJ/8), and a helper vector was prepared at a ratio of 1:4:2 and diluted in opti-MEM (Gibco, cat. #31985-070) containing PEI at a ratio of 2.5:1. The mixture was added to ~ 80% confluent HEK293T cells, cultured with FBS-free DMEM, and incubated for 4 h, after which the medium was replaced with FBS-supplemented DMEM. After 72 h, the medium and cells were harvested and centrifuged. The cells were digested with lysis buffer, whereas the supernatant was precipitated with 40% polyethylene glycol (PEG; Sigma, P2139). Cell lysates and precipitated substances were then mixed with sodium deoxycholate and benzonase, after which the mixture was subjected to freeze–thaw cycles and loaded onto an iodixanol gradient for ultracentrifugation at 350,000*g* for 1 h. Lastly, the 40% iodixanol layer was extracted and concentrated using an Amicon centrifugal filter (Merk, UFC910024).

### Reagents and hippocampal neuron culture and transfection

Human recombinant GDNF (Gibco, cat. #PHC7045) and ZCL278 (Tocris, cat. #4974) were prepared according to the manufacturers’ instructions.

Embryonic day 18 (E18) embryos were used for primary neuron culture. Briefly, after sacrificing pregnant Sprague–Dawley female rats, hippocampi from collected embryos were dissected in Hank’s balanced salt solution (Gibco, Cat. #14185-052) supplemented with 10 mM HEPES, 1 mM sodium pyruvate, 2 mM GlutaMAX (Gibco, cat. #35050-061) and 1% penicillin–streptomycin (P/S) solution. The collected hippocampi were digested with 0.25% trypsin for 15 min at 37 °C and then triturated with a fire-polished Pasteur pipette. The dissociated neurons were plated on 0.1 mg/mL poly-L-lysine pre-coated 24-well plates (Cellvis, cat. #P24-1.5H-N) or 50-mm dishes (MatTek, cat. #P50G-0–30-F) containing neurobasal medium (Gibco, cat. #21103-049) supplemented with 2% horse serum (Gibco, cat. #16050122), 2 mM GlutaMAX, and 1% P/S. After incubating cells at 37 °C in a humidified 5% CO_2_ incubator for 1 h, the medium was replaced with maintenance medium lacking horse serum but containing 2% B-27 (Gibco, cat. #17504-044).

Neurons plated on 24-well plates were transfected at 7 days in vitro (DIV7) using Lipofectamine LTX (Invitrogen, cat. #15338-100), according to the manufacturer’s instructions, and imaged on the following day. For experiments comparing AKT and ERK signaling, neurons were co-transfected with optoRTKs (optoRET, optoRET^D387A^ or optoTrkB [27]) and iRFP and mCherry-AKT-PH or ERK-KTR-FusionRed. For GDNF experiments, cells were co-transfected with optoRET^full−length^ and stimulated with GDNF (50 ng/mL). For actin structure experiments, neurons were co-transfected to express optoRET together with iRFP and mCherry-LifeAct. For inhibition of Cdc42 (cell division control 42), transfected neurons were pre-incubated with ZCL278 (100 µM) for 0.5 h prior to imaging.

### Confocal imaging and laser photoactivation

Confocal imaging was performed using a Nikon A1R confocal microscope mounted on an Eclipse Ti body (Nikon Instruments) equipped with a Plan Apochromat 20X objective (numerical aperture [NA], 0.75) or an Apochromat 60X oil objective (NA 1.4). Live-cell imaging was performed using a microscope stage-equipped Chamlide TC system (Live Cell Instruments) that maintains a 37 °C, 5% CO_2_ environment. A multi-line argon laser (405, 488, 561 and 647 nm), emitted through a Galvano scanner incorporated in a hybrid confocal scan head with a high-speed hyper selector (Nikon Instruments), was used for imaging and photoactivation.

For co-imaging photoactivation, the 488-nm laser was used to capture and stimulate the whole imaging field (512 × 512 µm) at 2-min intervals. For local photoactivation, regions of interest (ROIs) were illuminated with a sequence of 488-nm laser stimulations (5 times, without delay, for 2.39 s) at 1-min intervals. A 488-nm laser power of 22–24 µW/mm^2^ was used for both co-imaging and local photoactivations.

### Axotomy and photoactivation with light-emitting diodes (LEDs)

Axotomy experiments were performed using 50-mm dishes equipped with microfluidics chips (Zona, cat. #RD150). Prepared neurons were plated in a single compartment such that cell body and axonal sides were separated by a microgroove barrier as the neurons grew. On DIV4, AAV-hSyn1-optoRET(HA) and AAV-CamKIIα(0.4)-mScarlet-IRES2-CRE, diluted in fresh maintenance medium, were applied to the chip at final viral titers of 5 × 10^11^ and 5 × 10^12^ genomic copies/mL, respectively. On DIV11, the axonal sides of the chips were imaged and then axotomized by vacuum aspiration. After the axotomy, the axonal sides were imaged again to confirm that axons had been completely cut. For photoactivation, light was delivered using a customized blue LED array board (Live Cell Instruments) [36] with a customized plate-bottom cover that allows light to pass on the axonal side only. Light was delivered according to a defined on/off cycle (10-s on and 10-min off) at a light density of 25 µW/mm^2^ (488 nm). On DIV13, the axonal sides of the chips were imaged. Optimized LED photoactivation conditions were confirmed by additionally transducing neurons with AAV-CamKIIα-ERK-KTR-clover, visualizing the ERK activities induced by optoRET activation.

### Mice

Male DAT-CRE B6.SJL mice (The Jackson Laboratory, stock #006302), donated by Daesoo Kim at the Korea Advanced Institute of Science and Technology (KAIST), were used in this study. All mice were maintained under a 12-h light on/off cycle (light intensity measured at the center of the home cage, ~ 2 µW/cm^2^). Mice were group-housed with free access to food and water until subjected to stereotactic surgery at 10 wk of age.

### Stereotaxic surgery and transcranial photoactivation

Mice were anesthetized with 200 mg/kg 2,2,2-tribromoethanol (Avertin; Sigma, cat. #T48402) and their temperature was maintained at 37 °C by placing them on a surgery heating pad (Live Cell Instruments). After positioning mice in a stereotaxic device, a microdrill was used to perforate the skull above the target region (right SNc) with the following coordinates (relative to bregma): anterior–posterior, -3.6 mm; medial–lateral, + 1.3 mm; and dorsal–ventral − 3.5 mm. Using a 33-gauge blunt NanoFill needle (World Precision Instruments, cat. #NF33BL-2), 0.5 μL of AAV-hSyn1-DIO-optoRET (2 × 10^11^ genomic copies/mL) was injected into the target region at a flow rate of 70 nL/min.

After the surgery, mice were housed in pairs in a home cage, separated with a transparent separator. At 2 wk post-surgery, the home cage lid was replaced with a customized blue LED home cage lid for transcranial photoactivation [30]. For 1 wk, light (470 nm) was delivered for 5 h per day at a light density of 40 µW/cm^2^.

### Immunostaining

For the immunohistochemistry analysis, at 3 wk post-surgery, mice were anesthetized with 200 mg/kg Avertin and transcardially perfused, first with phosphate-buffered saline (PBS) and then with 4% paraformaldehyde (PFA). For photoactivated mice, the procedure was conducted after the light schedule had been finished. Brains were post-fixed in 4% PFA overnight and then coronally sectioned at a thickness of 50 µm using a vibratome (Leica, Cat. #VT1200S). Midbrain sections were collected and incubated in blocking solution, consisting of 5% normal donkey serum (NDS; Abcam, cat. #ab7475) and 0.3% Triton-X in PBS, for 1.5 h at room temperature (RT). Sections were immunostained by incubating overnight at 4 °C with the following primary antibodies (diluted in blocking solution): anti-phospho(S235/S236)-S6 ribosomal protein (pS6, 1:1000; Cell Signaling, cat. #4858), sheep anti-tyrosine hydroxylase (TH) (1:1000; Abcam, cat. #ab113) and mouse anti-HA (1:500; Cell Signaling, cat. #2367), the latter used for the detection of HA-tagged optoRET [optoRET(HA)]. Sections were subsequently stained by incubating for 1 h at RT with Alexa 488-conjugated anti-rabbit, Alexa 594-conjugated anti-sheep, or Alexa 647-conjugated anti-mouse secondary antibody, as appropriate, each at a 1:100 dilution. Sections were washed three times with PBS containing 0.3% Triton-X between steps. After the final wash, sections were mounted on cover glasses using a 4’,6-diamidino-2-phenylindole (DAPI)-containing mounting solution (Vector Laboratories, cat. #H-1200) and imaged with a confocal microscope.

For the immunocytochemistry, neurons were fixed in 4% PFA for 15 min at bench and then followed the same procedures as above. Mouse anti-ankyrin G (1:100, Santa Cruz, cat. #sc-12719) and donkey anti-mouse Alexa 405 (1:1000, Abcam, cat. #ab175658) antibodies were used.

### Western blot analysis

At 3 wk post-surgery, mice were anesthetized with 200 mg/kg Avertin, and their brains were extracted under a red-light lamp. For photoactivated mice, the procedure was conducted once the light schedule had finished. For each sample, the midbrain sections of the right and left side were separately collected in tubes and immediately snap-frozen in liquid nitrogen. The samples were then incubated in protein extraction solution (Pro-PREP; iNtRON, cat. #17081) containing a cocktail of phosphatase inhibitors (PhosSTOP; Sigma, cat. #4906845) for 20 min at − 20 °C. After centrifugation, the supernatant was transferred to new tubes and then denatured by boiling at 95 °C for 10 min in sodium dodecyl-sulfate polyacrylamide gel electrophoresis (SDS-PAGE) loading buffer (Biosesang, cat. #BIS-SF2002-110–00). The denatured proteins were resolved by SDS-PAGE on 4−12% Bolt Bis–Tris gels (Invitrogen, cat. #NW04125BOX) using Bolt MES SDS running buffer (Invitrogen, cat. #B0002). After running gels at 100 V for 1 h, proteins were transferred to a nitrocellulose membrane (Invitrogen, cat. #IB301001). The membrane was then blocked by incubating in Tris-buffered saline (TBS) blocking buffer (LI-COR, cat. #927059991) for 1 h at RT. After washing with TBS containing 0.1% Tween-20 (TBST), the membrane was incubated overnight at 4 °C with rabbit anti-phospho(Y202/Y204)-p44/42 MAPK (ERK1/2) (pERK1/2) primary antibody (Cell Signaling, cat. #9101S) and mouse anti-glyceraldehyde 3-phosphate dehydrogenase (GAPDH) antibody (Invitrogen, cat. #MA5-15,738), diluted 1:500 and 1:6000, respectively, in TBST. Membranes were then washed with TBST and incubated for 1 h at RT with goat anti-rabbit 680 RD (LICO, cat. #926-68071) or goat anti-mouse 800 CW (LICO, cat. #926-32210) secondary antibody, both diluted 1:10,000 in TBST. Membranes were then imaged using an Odyssey CLX imaging system (LICO, cat. #9140).

### Software and data analysis

All confocal images were processed and analyzed using Nikon NIS-elements AR imaging software (Laboratory Imaging, v.5.21). Skeleton images in axotomy experiments were analyzed using ImageJ software (USA National Institutes of Health, v.1.53t). Immunoblot images were processed and analyzed using Image Studio software (LICO, v.5.2). All graphs and heatmaps were plotted using Prism software (GraphPad, v.9.4), and schematic illustrations were created with tools available at the BioRender website (BioRender.com).

For the analysis of AKT activity using the biosensor AKT-PH, the intensities of cytosolic AKT-PH and volume control (iRFP) were measured using NIS software. The intensities at each time point were expressed as iAKT = (cytosolic AKT intensity)/(cytosolic iRFP). iAKT was converted to normalized cytosolic AKT-PH, expressed as nAKT = (iAKT)/(mean of the iAKT at the baseline, -10 to 0 min). nAKT was used for plotting graphs. AKT responsiveness to sustained stimulation of optoRET, optoRET^D387A^, optoTrkB, or GDNF was determined by measuring signal response, calculated as dAKT = (nAKT at 0 min)–(mean of the nAKT at 36 to 40 min). For the transient groups, the latter was the mean of the nAKT at 6–10 min. In cases where dAKT was greater than -0.15, the cell was scored as ‘True’ (100 points); otherwise, it was scored as ‘False’ (0 points). Activation half-life (T_1/2_) was determined by fitting nAKT in time graphs at 0–40 min using the one-phase decay equation in Prism software. For group comparisons in bar graphs, only values of T_1/2_ with successfully calculated 95% confident intervals (CIs) were used, and outliers identified using the ROUT (1%) method in Prism software were removed.

For the analysis of the ERK activity using the biosensor ERK-KTR, we measured intensities in the cytosol and the nucleus for the sensor and iRFP. iERK was calculated in the same manner as iAKT. The ratio of iERK in the cytosol to that in the nucleus (C/N) was calculated and expressed as rERK. rERK was then normalized, as was done for nAKT, and used for plotting graphs. To determine ERK responsiveness to sustained stimuli, we calculated dERK as we did for dAKT. For the transient groups, the latter was the mean of the nERK at 16–20 min. The cell was scored as ‘True’ (100 points) if dERK was greater than 0.15; otherwise, it was scored as ‘False’ (0 points). Activation T_1/2_ was determined following the same steps as used for AKT, except a one-phase association equation in Prism software was used for fitting.

For analysis of ERK activity levels, C/N ratios were calculated using the row intensities of ERK-KTR and then categorized into four different categories: low, k < 0.75; normal, 0.75 < k < 1.25; intermediate, 1.25 < k < 1.75; and high, 1.75 < k, where k = the C/N ratio. The frequency distribution of these data was used for plotting the ERK activity level heatmap.

For axotomy experiments, the ‘skeletonize’ tool in ImageJ software was used to convert row images to skeletonized images, which were used for the analysis. Axon lengths were measured with ImageJ software by drawing short lines on the terminal ends of axons and then manually counting the number of protrusions on the lines. The data were then converted to numbers of protrusions per 100 µm (protrusion densities) and categorized into three different axon morphological complexities: low, k < 10; intermediate, 10 < k < 30; and high, 30 < k, where k = protrusion density. The frequency distributions of these data were used for plotting the axon morphological complexity heatmap.

For the analysis of stained midbrain sections, the cell bodies of the TH-positive and pS6-positive cells were counted manually. The average of the Dark group was used for the normalization.

For the analysis of pERK1/2 protein levels, the intensities of bands at 42–44 MW and 38 MW were measured using Image Studio software and normalized to the intensity of the respective GAPDH bands. For each mouse, the pERK1/2 protein level was determined as the ratio of the right side (ipsilateral) to the left (contralateral) side and then normalized to the mean of the Dark group.

## Results

### Development of an optogenetic tool for modulating RET

**Fig. 1:**
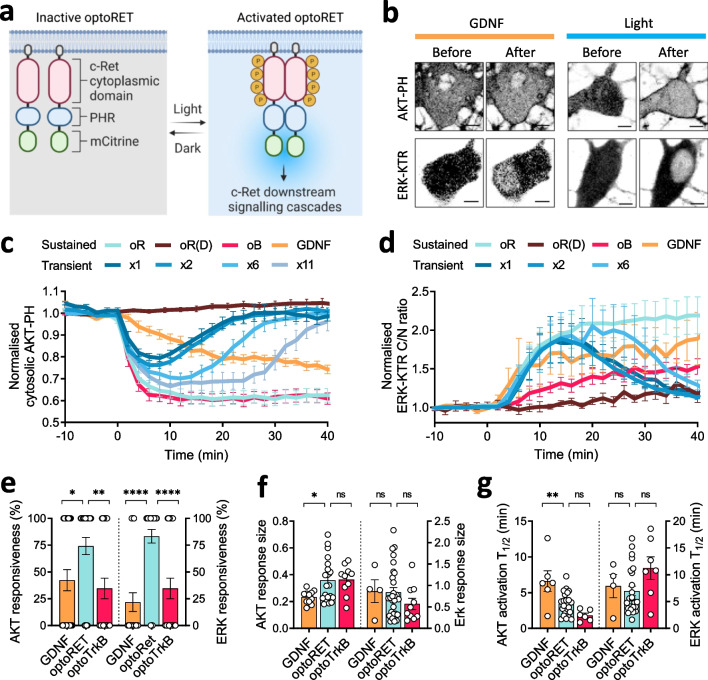
Development and characterization of optoRET. **a** Schematic diagram of optoRET. **b** Representative confocal images of cultured neurons, showing the localization of AKT-PH and EKR-KTR biosensors before and after sustained stimulation by GDNF (50 ng/mL) or light. Scale bars = 5 µm. **c**, **d** Graphs showing normalized cytosolic AKT-PH and ERK-KTR cytosol-to-nucleus (C/N) ratios as a function of time. Sustained stimulation was given at 0–40 min by illumination of neurons expressing optoRET (oR), optoRET_D387A_ [oR(D)] or optoTrkB (oB), or by application of GDNF to neurons expressing optoRET^full−length^ (GDNF). Different numbers of transient light stimulations were also administered to optoRET-expressing neurons, as follows: 1 stimulation at 0 min (× 1), 2 stimulations at 0–2 min (× 2), 6 stimulations at 0–10 min (× 6), and 11 stimulations at 0–20 min (× 11). For each group, n = 4–31 cells. **e** Comparison of AKT (left) and ERK (right) responsiveness to sustained stimulation with GDNF (AKT: n = 26; ERK: n = 23) optoRET (AKT: n = 31; ERK: n = 36), or optoTrkB (AKT: n = 26; ERK: n = 26). **f** Comparison of AKT and ERK response size for stimulation with GDNF (AKT: n = 11; ERK: n = 4), optoRET (AKT: n = 20; ERK: n = 28), or optoTrkB (AKT: n = 10; ERK: n = 10). **g** Comparison of AKT and ERK activation T_1/2_ following stimulation with GDNF (AKT: n = 6; ERK: n = 4), optoRET (AKT: n = 23; ERK: n = 24), or optoTrkB (AKT: n = 6; ERK: n = 7), obtained by fitting exponential curves. Data are presented as means ± SEM (*p < 0.05, **p < 0.01, ****p < 0.0001; one-way ANOVA); ns, not significant (p > 0.05)

Utilizing human RET9 and PHR^E281A^ [30], we designed two chimeric proteins for the optical modulation of RET signaling. For the first version, we attached the PHR tagged with mCitrine or HA to the C-terminus of full-length RET9 (aa 1–1972) and termed this chimeric protein, optoRET^full−length^ (Additional file 1: Fig. S1). The capacity to respond to endogenous RET ligands and co-receptors was preserved in optoRET^full−length^. For the second version, we utilized only RET-CYR (aa 658–1062), flanked by the myristoylation (Myr) domain of the Lyn signal peptide, and the PHR tagged with mCitrine or HA, and termed this chimeric protein, optoRET (Additional file 1: Fig. S1). The Myr domain was used for membrane localization of optoRET, replacing the RET-TMD. Thus, we generated two chimeric proteins that are empowered to respond to blue light (wavelength, 488 nm) through the homo-oligomerization property of the PHR. Upon light illumination, these proteins oligomerize, enabling tyrosine residues in RET-CYR to be trans-phosphorylated and activate downstream RET signaling cascades (Fig. 1a).

### Characterization of optoRET-induced AKT and ERK signaling

To examine the functionality of optoRET, we first tested whether light-induced oligomerization of optoRET could recruit Grb2, an adaptor protein of RET. To this end, we co-transfected cultured neurons with optoRET and Grb2-GFP, and then performed live-cell imaging using confocal microscopy, imaging Grb2-GFP and activated optoRET at 2-min intervals using a 488-nm laser (~ 25 µW/mm^2^). These experiments revealed robust clustering of Grb2-GFP at the soma and dendrites (Additional file 1: Fig. S2 and Additional file 2: Movie S1), indicating that photoactivation of optoRET successfully stimulated oligomerization-induced trans-phosphorylation of its tyrosine residues and then recruited its adaptor protein, Grb2, into multiple clusters of signaling condensates.

Because Grb2, as a downstream effector of RET, is among the adaptor proteins that mediate PI3K/AKT and Ras/MAPK pathways (Additional file 1: Fig. S1), we examined the activation of AKT and ERK signaling by light. Multiple MAPKs, including ERK1/2, ERK5, JNK and p38MAPK, are reported to be downstream targets of RET; as the most frequently reported such target, the ERK family was selected as the readout for Ras/MAPK pathway activity [37–39]. AKT and ERK signaling activities were visualized using two genetically encoded biosensors: the pleckstrin homology domain of AKT1 (AKT-PH) [33, 40] conjugated to the C-terminus of mCherry, and the ERK kinase translocation reporter (EKR-KTR) [35] conjugated to FusionRed, respectively. The shuttling of mCherry-AKT-PH from the cytosol to the plasma membrane reflects the translocation of AKT upon PI3K-mediated phosphorylation of phosphatidylinositol 4,5-bisphosphate (PIP_2_) to phosphatidylinositol 3,4,5-bisphosphate (PIP_3_), providing an indicator of AKT signaling activity [33, 40]. Because ERK-KTR-FusionRed, comprising the ERK docking site of ETS Like-1 protein (Elk1) and phosphorylation sites, shuttles from the nucleus to the cytosol when phosphorylated by ERK, ERK-KTR-FusionRed fluorescence reflects ERK kinase activity [35]. Thus, the clearer the plasma membrane pattern of mCherry-AKT-PH and nuclear pattern (high C/N ratio) of ERK-KTR-FusionRed, the higher AKT or ERK signaling activities, respectively.

Cultured neurons were co-transfected with optoRET, iRFP and mCherry-AKT-PH or ERK-KTR-FusionRed, and then live-cell imaging was performed using confocal microscopy. Biosensors were imaged before and after sustained light stimulation for 40 min at 2-min intervals using the co-imaging photoactivation protocol employing a 488-nm laser (~ 25 µW/mm^2^). We also performed the same experiment with GDNF (50 ng/mL), except that neurons were transfected with optoRET^full−length^ because hippocampal neurons are reported to only endogenously express GFRα and not RET [3, 41, 42]. A comparison of the intensities of mCherry-AKT-PH in the cytosol and ERK-KTR-FusionRed in the nucleus before and after light or GDNF (50 ng/mL) stimulation showed clearly distinguishable plasma membrane or nuclear patterns after the respective stimulations (Figs. 1b and Additional file 1: Fig. S3a, b).

For additional quantitative and qualitative comparisons, we performed the same experiment on neurons expressing a light-insensitive form of optoRET [optoRET^D387A^, oR(D)] or optoTrkB (oB), another PHR-based optoRTK tool that modulates TrkB signaling [27] (Figs. 1c, d and Additional file 1: Fig. S3c). As expected, we found that none of the cells in the optoRET^D387A^ group showed AKT or ERK responsiveness (see, *Methods* for details). Thus, in subsequent analyses, we only compared sustained GDNF, optoRET, and optoTrkB groups.

First, we found that the proportions of cells showing AKT or ERK signaling responsiveness were much lower in GDNF or optoTrkB groups, compared to optoRET group (Fig. 1e**)**. For AKT, the GDNF, optoRET and optoTrkB groups had 42.31 ± 9.88% (n = 26), 74.19 ± 7.99% (n = 31) and 34.62 ± 9.52% (n = 26), respectively. For ERK, the optoRET group was 83.33 ± 6.30% (n = 36), which were far beyond those for the GDNF and optoTrkB groups (21.74 ± 8.79%, n = 23; 34.62 ± 9.52%, n = 26; *p* > 0.0001).

In terms of the absolute values of response magnitudes (response size), there were no noticeable differences in ERK signaling among GDNF (0.87 ± 0.27%, n = 4), optoRET (0.84 ± 0.11%, n = 28) and optoTrkB (0.57 ± 0.13%, n = 10) groups (Fig. 1f, right bar graphs). In contrast, the response size for AKT signaling was significantly smaller for the GDNF group (0.23 ± 0.01%, n = 11; *p* < 0.05) compared with the optoRET group (0.36 ± 0.04%, n = 20), but was comparable between the optoTrkB group and optoRET group (0.36 ± 0.04%; n = 10) (Fig. 1f, left bar graphs).

Because light-mediated activation of RTKs bypasses steps involving ligand interactions with the extracellular domain of the receptors, the kinetics of downstream signaling activation is likely to be faster than that for ligand-mediated activation. To test this, we compared AKT and ERK activation kinetics (T_1/2_) among GDNF, optoRET, and optoTrkB groups in the sustained stimulation experiments (Additional file 1: Fig. S4 and Fig. 1g). The activation kinetics for AKT were significantly longer in the GDNF group (6.63 ± 1.44 min, n = 6; *p* < 0.001), compared with the optoRET group (3.44 ± 0.33 min, n = 23), while that for the optoTrkB group (1.81 ± 0.32 min, n = 6) was not significantly different compared with the optoRET group (Fig. 1g, left bar graphs). Interestingly, there were no apparent differences in ERK activation kinetics among GDNF (7.89 ± 2.16 min, n = 4), optoRET (6.99 ± 0.89 min, n = 24) and optoTrkB (11.24 ± 2.09 min, n = 7) groups (Fig. 1g, right bar graphs). Collectively, these results indicate that photoactivated optoRET recruits Grb2 into clusters and induces AKT and ERK signaling activity with fast activation kinetics for AKT and robust responsiveness for ERK signaling.

### Dynamic control of AKT and ERK signaling by light

It has been shown the dynamic control of the input signal is an important factor that determines the resultant cellular response in many biological processes [43, 44]. Because optoRET can toggle between active and inactive states upon association and dissociation of the conjugated PHR protein, varying the duration of the photoactivation could enable the dynamic control of AKT and ERK signaling activities. Therefore, we examined AKT and ERK signaling activity in response to different numbers of light stimulations of neurons expressing optoRET, iRFP and mCherry-AKT-PH or ERK-KTR-FusionRed (Fig. 1c, d, transient groups). Based on the reported time constant for PHR dissociation (~ 5.5 min) [45], our co-imaging photoactivation protocol could maintain optoRET in a continuously activated state throughout the intervening periods between stimulations. Thus, our manipulation of the number of light stimulations can be considered the equivalent of varying the duration of the photoactivation.

We first tested if a single stimulation at 0 min (transient, × 1) was able to induce AKT or ERK signaling activity. Interestingly, single stimulation was sufficient to elicit both AKT and ERK signaling (Fig. 1c, d, transient, × 1), although the responsiveness were significantly lower than sustained stimulations (Additional file 1: Fig. S5a). Increasing the number of light stimulations to 2 (0–2 min), 6 (0–10 min) or 11 (0–20 min) increased the magnitudes of AKT activity (Fig. 1c and Additional file 1: Fig. S5b left). On the other hand, there were no apparent differences in the magnitude of response size of ERK signaling between transient groups and sustained optoRET (0–40 min) group (Fig. 1d and Additional file 1: Fig. S5b right). AKT and ERK signaling recovered in transient groups after having reached the maximal responses (Fig. 1c, d). Taken together, our results indicate that, by modulating the duration of the photoactivation, optoRET can dynamically regulate AKT and ERK signaling.

### Retrograde signal transmission of distal optical input to the cell body of a neuron

One of the great advantages of optogenetics is the high spatial resolution achievable using a light stimulus, which allows us to modulate specific signaling at specific subcellular levels in a highly controllable manner. In line with this, subcellular, local photoactivation of various optoRTKs has deciphered many cellular signaling processes, including directed migration (with optoFGFR1 [25]) and determination of axonal fate (with optoTrkB [27]). Interestingly, optoRTKs also undergo a receptor internalization process following photoactivation and then are retrogradely transported, resulting in transmission of the optical input from the stimulated region to the cell body [27]. Accordingly, we hypothesized that local optical input on a distal part of an optoRET-expressing neuron could be retrogradely transduced to activate AKT or ERK signaling in its cell body, as is the case for active RET [46, 47].

**Fig. 2:**
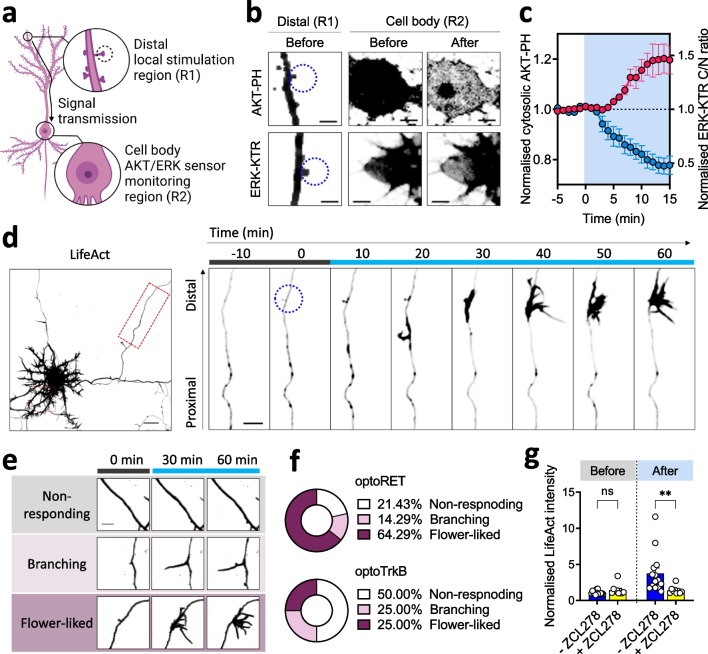
Local stimulation of optoRET in cultured neurons. **a** Schematic diagram of a neuron, highlighting retrograde signal transmission from a distal, local region (R1) to the cell body (R2). The R1 constitutes a local photoactivated area, indicated by the dashed circle, and R2 is where the activities of AKT and ERK biosensors are monitored. **b** Representative confocal images of R1 and R2 in locally photoactivated neurons expressing optoRET and biosensors of AKT or ERK activity before and after photoactivation. The illuminated areas are indicated by dashed blue circles. Scale bars = 5 µm. **c** Quantification of AKT and ERK activation in the cell bodies of neurons by distal, local optoRET stimulations (0–15 min). Data are presented as means ± SEM (AKT in blue, n = 7; ERK in red, n = 5). **d** Representative confocal images of a neuron expressing optoRET and an F-actin biosensor (LifeAct). The cropped area (dashed red rectangle) is highlighted in time-lapse images on the left, and the photoactivated area is indicated by the dashed blue circle in the image at 0 min. Scale bars = 20 and 10 µm. **e** Representative time-lapse confocal images of LifeAct, corresponding to the three classes of responses to the local photoactivation of optoRET before (0 min) and after (30 and 60 min) photoactivation. Scale bar = 10 µm. **f** Comparison of the proportions of responses in non-responding, branching and flower-like structural reorganizations, induced by local photoactivation of optoRET (n = 14) or optoTrkB (n = 8). **g** Comparison of normalized LifeAct intensities before (0 min) and after (60 min) local photoactivation of optoRET, in the absence (blue, n = 14) or presence (yellow, n = 9) of the Cdc42 inhibitor, ZCL278 (100 µM; 0.5 h pre-incubation). Data are presented as means ± SEM (**p < 0.01; two-way ANOVA); ns, not significant (p > 0.05)

To test our hypothesis, we co-transfected cultured neurons with optoRET and mCherry-AKT-PH or ERK-KTR-FusionRed, together with iRFP, and performed live-cell imaging on the cell bodies (R2) of the transfected neurons while locally photoactivating overexpressed optoRET on distal regions (R1; laid on axons, 227 ± 18.65 µm distance from soma) of the neurons (Fig. 2a). These experiments revealed translocation of the biosensors into the cell bodies (R2) after local photoactivation of distal regions (R1) of the neurons (Figs. 2b and Additional file 1: Fig. S6a, b). A quantitative analysis of biosensors in R2 showed that AKT and ERK signaling activities started to increase from their initial levels prior to photoactivation (0 min) after a few minutes of a static phase (Figs. 2c and Additional file 1: Fig. S6). Interestingly, this static phase was not evident in experiments using the previous co-imaging photoactivation protocol, where increases in AKT and ERK signaling activities were observed immediately after the initial photoactivation at 0 min (Fig. 1c, d). We investigated any potential correlations between the stimulated ROI distance from the soma and the duration of the static phase. The Pearson’s correlation (r) was only 0.14, suggesting no significant correlations (Additional file 1: Fig. S6c). Additionally, we verified that the target neurites were indeed axons by staining Ankyrin G (Additional file 1: Fig. S6d), which is reported to be present in axons [27]. These results demonstrate that local activation of optoRET on a distal part of axon retrogradely transmits the local input to the soma for the activation of AKT and ERK signaling.

### Formation of filopodia-like structures by local photoactivation

As is the case for many other neurotrophic receptors, activation of RET can also promote branching morphogenesis and neurite outgrowth of neurons [17, 18, 20, 27, 48, 49]. In line with previous studies, we also observed structural changes following local photoactivation of optoRET (Additional file 1: Fig. S6a). To further evaluate these results, we performed local photoactivation experiments on neurons expressing optoRET and the F-actin biosensor, mCherry-LifeAct [25, 34].

Surprisingly, local photoactivation on axons induced a large accumulation of F-actin around the stimulated area followed by robust formation of filopodia-like structures (Fig. 2d). Time-lapse images revealed an increase in anterogradely transported F-actin, initiating at the proximal side of the photoactivated area and propagating to the stimulated area (Fig. 2d). After 30-min of photoactivation, a large accumulation of the F-actin was observed at the stimulated area, after which they reorganized, forming many filopodia-like structures (Fig. 2d and Additional file 3: Movie S2). We termed this type of newly formed structure, ‘flower-like’, reflecting the fact that the process that gave rise to such structures resembled that of a blooming flower (Additional file 1: Fig. S7).

Local photoactivation-induced structural reorganization occurred at ~ 80% of stimulated regions (n = 11/14), and more than 80% (n = 9/11) of the responding regions showed formation of the flower-like structures, with the remainder forming simply branched 1–2 filopodia-like structures (Fig. 2e, f). Given that light-induced activation of optoTrkB was reported to promote local filopodia formation [24], we investigated how TrkB- and RET-mediated regulation in this respect differed. Thus, we performed the same experiment on neurons expressing optoTrkB in place of optoRET and found that the formation of flower-like structures was not as efficient as with optoRET (Fig. 2f). Despite keeping all experimental conditions constant except for the optoRTK construct used, only 25% (n = 2/8) of ROIs showed formation of flower-like structure (Fig. 2f).

Cdc42 is a well-known small GTPase of the Ras homologous (Rho) family that regulates F-actin polymerization into a filopodia-like structure [27, 50]. Accordingly, we assumed that light-induced F-actin structural reorganization elicited by stimulation of RET signaling cascades was mediated by downstream activation of Cdc42. To test this supposition, we performed the same experiments in the presence of the Cdc42 inhibitor, ZCL278 (100 µM; 0.5 h pre-incubation). These experiments showed that filopodia-like structures failed to form in the presence of the inhibitor (Additional file 1: Fig. S8). An analysis of normalized LifeAct intensities in ROIs, each comprising a photoactivation area, for the two groups revealed that more than 90% of ROIs (n = 13/14) in the group without ZCL278 showed an increasing trend of LifeAct intensities (Additional file 1: Fig. S8b). In contrast, ~ 90% of ROIs (n = 8/9) in the group with the inhibitor showed no such increasing trend (Additional file 1: Fig. S8c). In addition, intensities were increased in three ROIs (one in the with inhibitor group and two in the without inhibitor group) in the absence of newly formed filopodia-like structures. On average, the difference in the normalized intensities of LifeAct between the two groups was not significant before (at 0 min) photoactivation of ROIs (Fig. 2g, left bar graphs). However, after 1 h of photoactivation, the photoactivation-induced enhancement of the LifeAct intensities at ROIs was significantly blocked in the presence of ZCL278 (*p* < 0.001) (Fig. 2g, right bar graphs, and Additional file 1: Fig. S8d). Taken together, these results demonstrate that local photoactivation of optoRET induces an accumulation of F-actin, accomplished by the recruitment of anterogradely trafficked F-actin, after which structural reorganization of actins occurs through Cdc42 activity.

### Optical enhancement of sprouting protrusions on regenerating axons

In addition to the neuroprotective effects of RET signaling in various types of neurons, neuroregenerative effects are also well-established by previous studies [1–3, 23, 51]. In particular, it has been shown that RET signaling is essential for the regeneration of damaged axon terminals of DA neurons [23, 52]. In this context, we tested whether optical activation of optoRET would enhance the regeneration of damaged axons.

**Fig. 3:**
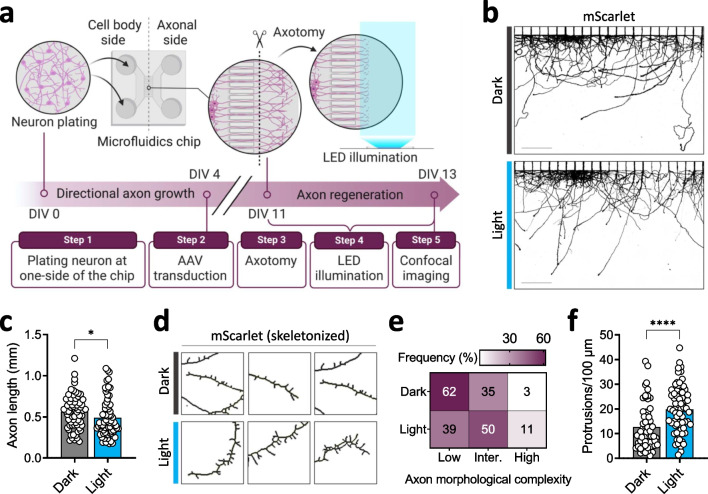
Optically enhanced formation of protrusions on regenerating axons after axotomy. **a** Schematic diagram of the axotomy experimental scheme. Neurons were loaded in the left compartment (cell body side) and axons grew to the right compartment (axonal side) through the microgroove barrier. On DIV4, neurons were transduced with AAVs expressing mScarlet and optoRET. On DIV11, neurons underwent axotomy and axons were allowed to regenerate for two consecutive days, during which the axonal side of the chip was illuminated with a blue LED plate. On DIV13, the axonal side of the microfluidics chip was imaged. **b** Representative confocal images of the axonal sides of microfluidics chips on DIV13. Scale bars = 200 µm. **c** Comparison of regenerated axon length (mm) between Dark (n = 64) and Light (n = 70) groups. Data are presented as means ± SEM (*p < 0.01; Kolmogorov–Smirnov test). **d** Representative skeletal images of regenerated axons. **e** Heatmap of the frequency distribution of low, intermediate, and high classes of axon morphological complexity. **f** Comparison of protrusion densities (protrusion count/µm) between Dark (n = 61) and Light (n = 69) groups. Data are presented as means ± SEM (****p < 0.0001; Kolmogorov–Smirnov test)

To this end, we took advantage of a microfluidics chip that enabled us to culture neurons in two compartments: one for the cell bodies and another for their axons. We loaded the prepared neurons in the soma compartment (cell body side) such that their axons could grow into the axonal compartment (axonal side) through a microgroove barrier connecting the two compartments (Fig. 3a). On DIV4, we transduced neurons with AAVs expressing mScarlet and optoRET (Additional file 1: Fig. S8a).

In parallel, we optimized the photoactivation protocol for this experiment using a customized blue LED array board (Live Cell Instruments) [36]. As a readout, we co-transduced neurons with AAV constructs expressing ERK-KTR and analyzed the C/N ratio of the biosensor. In the absence of photoactivation (Dark, n = 109), ~ 60% of neurons had a C/N ratio between 0.75 and 1.25, which we termed the normal ERK activity range (Additional file 1: Fig. S9b,c). Upon LED photoactivation (Light, n = 61), about half of the neurons with a normal ERK activity level shifted to a higher level (Additional file 1: Fig. S9b,c). Overall, our optimized LED photoactivation protocol was efficient enough to significantly increase ERK activity levels in transduced neurons (Dark group, 1.25 ± 0.03; Light group, 1.41 ± 0.04; *p* < 0.0001; Additional file 1: Fig. S9d).

On DIV11, we double-checked the axonal side of the microfluidics chip before and after axotomy of the transduced neurons to ensure the procedure successfully cut the axons (Additional file 1: Fig. S9e). Thereafter, we allowed the axons of neurons to regenerate for two consecutive days (recovery phase) before imaging the axons on DIV13. During the recovery phase, we illuminated the axonal side of the neurons for the photoactivated (Light) group using the optimized LED photoactivation protocol. Unexpectedly, we found that the length of regenerated axons was reduced by ~ 10% in the Light group (0.49 ± 0.03 mm, n = 70) compared with the Dark group (0.56 ± 0.26 mm, n = 64; *p* < 0.01; Fig. 3b,c).

Interestingly, however, the protrusion density (number of protrusions/100 µm) of regenerated axons in the Light group appeared higher than those in the Dark group (Fig. 3d). To quantify this, we classed axon morphological complexity into three categories according to protrusion density—low, intermediate, and high—with ranges of < 10, 10–30, and > 30, respectively (Additional file 1: Fig. S8f). Using this approach, we found that the major axon morphological complexity class for the Dark group was low (63%, n = 61), while that for the Light group was intermediate (50%, n = 69; Fig. 3e). This one-sided shift in the distribution frequency was reflected in the significantly increased protrusion density for the Light group (19.86 ± 1.15) compared with the Dark group (12.62 ± 1.16; *p* < 0.0001; Fig. 3f). Collectively, these results indicate that, despite a minor decrease in axonal length, optical activation of optoRET enhances protrusion sprouting on regenerating axons.

### Transcranial photoactivation of optoRET in the mouse brain

**Fig. 4:**
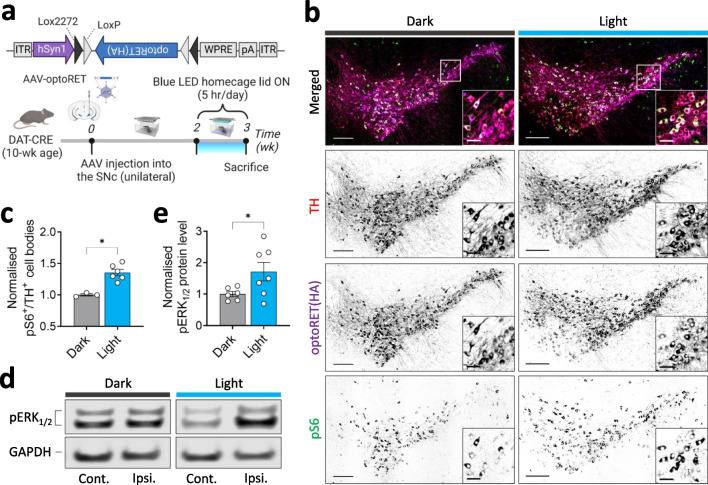
Transcranial optoRET activation in a mouse brain using a blue LED home cage lid. **a** Schematic diagram of the experimental scheme for optoRET activation in vivo. **b** Representative immunohistochemistry images of the SNc regions of mice expressing optoRET, with (Light group) or without (Dark group) LED illumination. Scale bars = 200 and 50 µm (cropped image). **c** Comparison of pS6-positive TH-stained cell counts in the SNc region of mice between Dark (n = 3) and Light (n = 6) groups. Data are presented as means ± SEM (*p < 0.05; Kolmogorov–Smirnov test). **d** Representative images of immunoblots for pERK1/2 and GAPDH. Protein samples were prepared separately for the virus-injected (ipsilateral) side (Ipsi.) and un-injected (contralateral) side (Cont.) of mice expressing optoRET. **e** Comparison of pERK1/2 protein levels between Dark (n = 6) and Light (n = 7) groups. pERK1/2 protein levels are expressed as ipsilateral relative to contralateral (pERK1/2/GAPDH), and data are presented as means ± SEM (*p < 0.05; Kolmogorov–Smirnov test)

Because RET is highly expressed in DA neurons of the SNc [1, 3], we sought to use optoRET to modulate RET signaling in these neurons in mice. For DA neuron-specific expression in the brain, we utilized a DAT-CRE transgenic mouse line that expresses CRE recombinase under control of the dopamine transporter (DAT) promoter, which allows floxed alleles to be expressed specifically in DA neurons, and injected AAVs expressing optoRET into the right SNc region of the mouse (Fig. 4a). Two weeks after AAV injection, we subjected freely moving mice to transcranial photoactivation for 1 wk (40 µW/mm^2^, 5 h/d) in their home cage, equipped with a customized blue LED lid (Fig. 4a) [30].

The ability of blue LED light to penetrate through the fur and skull of mice had previously been studied in depth [53], and the penetrated light was shown to be sufficient to induce PHR oligomerization, and thus downstream signaling of the PHR-based optogenetic tool, monSTIM1 [30]. Based on results obtained in retrograde transmission experiments in Fig. 2a-c, we hypothesized that light that penetrated in the dorsal striatum could activate optoRET that had been expressed in the axon terminals and that this optical input would be transmitted to produce activation of downstream signaling in the cell bodies located in the SNc.

Three weeks after AAV injection, mice were sacrificed and midbrain sections were prepared for immunohistochemical (IHC) detection of TH, optoRET(HA), and pS6. Imaging of stained samples revealed that optoRET was highly co-localized with TH-positive neurons, confirming the highly DA neuron-specific expression of optoRET (Fig. 4b). We further compared pS6-positive populations of TH-positive neurons in the SNc between mice without (Dark, n = 3) and with (Light, n = 6) transcranial photoactivation (Fig. 4b,c**)**. We found that photoactivation of optoRET significantly increased the proportion of pS6/TH double-positive neurons (Dark group, 1.00 ± 0.02; Light group 1.35 ± 0.05; *p* < 0.05) (Fig. 4b,c).

We also prepared the midbrains of mice for Western blot analysis of pERK1/2 and GAPDH expression (Fig. 4d). An assessment of immunoblot images showed that transcranial photoactivation of optoRET significantly increased pERK1/2 protein levels (Dark group, 1.00 ± 0.08, n = 6; Light group, 1.71 ± 0.29, n = 7; *p* < 0.05; Fig. 4d,e). Taken together, these results demonstrate that transcranially photoactivated optoRET induces signaling downstream of RET in DA neurons of the SNc in mice.

## Discussion

In this study, we developed a new optogenetic tool, termed optoRET, that modulates RET signaling. Using a control optoRET engineered to contain only RET-CYR, we eliminated the possibility that endogenous RET ligands and co-receptors could activate this chimeric protein [2, 4, 11]. Thus, RET-related functions in various biological systems can be studied using optoRET, regardless of the complex relationships among the five different endogenous RET ligands and co-receptors (Additional file 1: Fig. S1). Elimination of the RET-EXR required replacing the RET-TMD with the Lyn signal peptide; this modification was made necessary by the need to minimize basal activity, reflecting the reported strong self-association property of the RET-TMD [2, 11, 55]. Although this property might not directly result in the trans-phosphorylation of RET, it could result in consecutive activation of RET signaling in the absence of the WT RET-EX, as supported by results of mutagenesis studies and the prevalence of mutations in multiple endocrine neoplasia type 2A (MEN2A) [2, 11, 55].

Experimentally, we demonstrated the ability of optically activated optoRET to recruit Grb2 into multiple clusters of signaling condensates and activate AKT and ERK signaling (Additional file 1: Fig. S1, S2). A comparison of AKT and ERK signaling induced by optoRET, GDNF and optoTrkB revealed that optoRET was the most efficient in activating ERK signaling, exhibiting very high responsiveness in cultured neurons (Fig. 1e). Indeed, our results suggest that optoRET is capable of fully activating ERK signaling downstream of RET. By comparison, the concentration of GDNF used in these experiments was apparently insufficient to fully activate ERK signaling, as these cells showed further responses upon subsequent photoactivation (our unpublished data). Recent structural studies have provided evidence that RET ternary complexes not only exist as dimers but also as much high-ordered oligomers [11]. Because optical activation of optoRET primarily produces an oligomeric state, it might be reasonable to speculate that related signaling molecules, including adaptor proteins, are more concentrated locally in the optically activated scenario than with ligand activation. Mechanistic studies of RTKs have highlighted the emerging role of liquid–liquid phase separation at the plasma membrane in enhancing activation of signaling downstream of RTKs [56]. Consistent with this, it is possible that oligomerized optoRET induces more efficient RET downstream signaling than GDNF as a result of the increased probability of the required signaling molecules interacting with RET-CYRs through recycling in the active zone. The fact that this mechanism is likely less effective for the PI3K/AKT pathway might explain why ERK signaling in response to optoRET activation was dramatically enhanced compared with that of AKT. However, it is worth noting that this strong ERK signaling is bestowed by genuine properties of RET-CYR through the recruitment of multiple Ras/MAPK pathways [2, 6, 20, 38, 39], as we did not observe such dramatic ERK responsiveness with optoTrkB (Fig. 1e).

Our investigations of the dynamic control of AKT and ERK signaling revealed another characteristic property of these RET downstream signaling effectors. Varying the duration of the photoactivation showed that AKT signaling was positively correlated with the AKT response size, whereas that of ERK did not (Fig. 1c,d, Additional file 1: Fig. S5). These observations may reflect a characteristic property of RET modulation of ERK signaling, as evidenced by the fact that the duration of optical input was also shown to be positively correlated with the ERK response size in optogenetic experiments using optoFGFR [25] or optoTrkB [24]. However, these latter studies employed non-neuronal cells; thus, further characterization is needed to conclude that this observation regarding ERK signaling properties is solely mediated by the RET-CYR.

Another interesting point in comparisons of AKT and ERK signaling relates to differences in ligand-mediated (GDNF group) and light-mediated (optoRET or optoTrkB) activation kinetics. Although we expected that activation of ATK and ERK signaling by light would be faster than by a ligand, this was only the case for AKT signaling (Fig. 1g). We speculate this is because ERK translocation requires multiple phosphorylation steps (long-range signaling pathway), while AKT translocation involves only a single phosphorylation step (short-range signaling pathway). Thus, the more rapid activation of receptors by optical input might have been buffered by the multiple phosphorylation steps in the long-range signaling pathway. In addition, the sequential recruitment of adaptor proteins might have exaggerated the difference in AKT activation kinetics. Ligand-mediated activation of signaling is generally initiated in a lipid raft, with subsequent signaling occurring outside this microdomain [2, 21]. Previous studies have reported that RET-CYR forms a complex with FRS2 in rafts for Ras/MAPK signaling and then is translocated out of rafts, where it recruits Shc to mediate PI3K/AKT signaling cascades [2, 21]. On the other hand, active optoRET can complex with Shc outside of rafts and concomitantly with FRS2 in the raft immediately after the optical input is applied. This property of light-mediated activation might have accelerated the activation kinetics of AKT.

In the study of retrograde signaling transmission in Fig. 2a-c, we observed a few minutes of delay to response in soma. One of the possibilities to explain the static phase between the optical input and signal output at the soma may be an involvement of the process of internalizing active optoRET into a retrograde signaling endosome that transmits the local input to the soma [46, 47, 57]. According to a study that tracked quantum dot-labeled nerve growth factor (NGF), a dimeric NGF-bound TrkA-containing endosome can travel from a distal axon to the soma at a speed of ~ 1.3 μm/s [58]. In other words, a signaling endosome could travel ~ 400 μm in 5 min. This time frame seems compatible with the idea that active optoRET is internalized and physically travels back to the soma during the observed static phase. A previous study reported that internalized GDNF-activated RET could be subjected to proteosome-mediated degradation or retrogradely transported to the soma to relay the GDNF survival signal [47]. In this context, the demonstration that optoRET retrogradely transmits signals downstream of RET supports the feasibility of using this tool as a survival-promoting factor for neurons, although the mechanism should be further studied.

Our local stimulation experiments using LifeAct demonstrated the robust formation of filopodia-like protrusions by optoRET that were restricted to the photoactivated areas (Fig. 2d). The efficiency of optoRET in this process was almost double that of optoTrkB, and this profound F-actin structural reorganization induced by optoRET was mediated by Cdc42 activity (Fig. 2e-g). These observations seem to suggest that multiple signaling pathways downstream of RET favor the formation of filopodia-like structures. First, pY687 in the RET-CYR is reported to inhibit Rac1, which mediates the formation of lamellipodia [59]. Second, multiple adaptor proteins that serve as downstream effectors of RET are reported to promote neurite outgrowth, including SH2-Bβ [17], DOKs [18, 20], and Grb2 [2]. In particular, the recruitment of DOK4/5 promotes neurite outgrowth and branching through sustained ERK1/2 activation [20]. In line with this, it is possible that there are uncharacterized ERK-mediated mechanisms downstream of RET that promote Cdc42 activity, given our observation of significantly elevated efficiency of optoRET in activating ERK signaling (Fig. 1e). Interestingly, a recent study suggested dual roles for ERK3, an atypical MAPK, reporting that it acts as a guanine nucleotide exchange factor (GEF) for Cdc42 and also as a kinase for the actin-related protein 3 (ARP3) to promote filopodia formation and F-actin polymerization, respectively [50]. Although there is no direct evidence that RET regulates ERK3, it is reasonable to speculate that ERK3 could participate in a positive feedback loop in the activation of Cdc42 because one of the downstream targets of Cdc42, p21-activated kinase (PAK), is an ERK3 activator [50]. As a possibility for future work in this area, one approach to understand the underlining mechanism of optoRET-induced flower-like protrusions would be mutagenesis studies on the various tyrosine residues, involved in the RET signaling cascades (Additional file 1: Fig. S1).

We further demonstrated a profound enhancing effect of activated optoRET on the formation of protrusions on regenerating axons (Fig. 3). Specifically, we found that activated RET signaling enhanced the morphological complexity of regenerating axons rather than causing them to grow longer. This observation is reminiscent of the previously reported effect of RET on the regeneration of DA axon terminals in a PD mouse model [52] and might suggest an underlying mechanism of this previous observation. The axon terminals of DA neurons are extensively ramified, with millions of synapses in the striatum [54]; thus, optical enhancement of morphological complexity with optoRET could favor restoration of the functionality of the surviving axon terminals of DA neurons in PD mice.

Lastly, and importantly, we also successfully modulated signaling downstream of RET in mice by transcranially activating optoRET. Despite the well-established benefits of GDNF/RET signaling in the treatment of PD, modulating this signaling as a therapeutic intervention has not proven to be straightforward [54, 60, 61]. In previous studies, prolonged overexpression or long-term exposure of DA neurons to GDNF did not efficiently activate RET signaling and even resulted in the aberrant branching of axons [60, 62]. In this context, many researchers have attempted to develop controllable systems for modulating RET signaling in vivo [28, 63, 64]. In the current study, we first demonstrated optical control of RET signaling in DA neurons of the SNc in a mouse brain. Because the light-delivery procedure was non-invasive and finely controllable over time, and the subject mice could freely move in their home cage, it is possible that optoRET could be developed as a future therapeutic intervention for treating various neurological disorders, such as PD.

In conclusion, we present a new optogenetic tool, optoRET, for regulating RET signaling that shows high spatiotemporal regulation in cultured neurons and mice. By comparing optoRET with other optoRTKs, we demonstrated that optoRET preserves the properties of endogenous RET in activating downstream signaling. We established optoRET as a robust activator of ERK signaling and enhancer of the morphological complexity of axons, and successfully applied this tool to modulate RET downstream signaling in DA neurons of the SNc region in mice. Our application of optoRET in the mouse brain opens the possibility of using this tool to treat various neurological disorders.

## Supplementary Information

**Additional file 1: Figure S1.** Comparison of the structures of RET and its chimeric variants used for transduction of downstream signaling pathways. **Figure S2.** Robust clustering of Grb2 induced by activation of optoRET. **Figure S3.** Comparison of AKT and ERK signaling activation by GDNF, optoRET, and optoTrkB. **Figure S4.** Exponential curve fitting of AKT and ERK signaling activation. **Figure S5.** Comparison of the effect of dynamic stimulation of optoRET. **Figure S6.** Retrograde AKT and ERK signal transmission by optoRET. **Figure S7.** Flower-like F-actin structural reorganizations induced by the local photoactivation of optoRET. **Figure S8.** Inhibition of Cdc42 blocks the photoactivated F-actin structural reorganization. **Figure S9.** Axotomy of cultured neurons and optoRET activation with blue LED illumination.

**Additional file 2: Movie S1.** Photoactivated optoRET recruits its downstream signaling molecule, Grb2.

**Additional file 3: Movie S2.** Optical enhancement of F-actin structural reorganization by optoRET.

## Data Availability

The data that support the findings of this study are available from the corresponding author upon reasonable request.

## Abbreviations

RET: REarranged during Transfection
Grb: Growth factor receptor-bound protein
ERK: Extracellular signal-regulated kinase
Cdc42: Cell division control 42
RTK: Receptor tyrosine kinase
DA: Dopaminergic
EXR: Extracellular region
TMD: Transmembrane domain
CYR: Cytoplasmic region
CLD: Cadherin-like domain
CRD: Cysteine-rich region
JMD: Juxtamembrane domain
TKD: Tyrosine kinase domain
aa: Amino acid
GDNF: Glial cell line-derived neurotrophic factor
GFL: GDNF family ligand
NRTN: Neurturin
ARTN: Artemin
PSPN: Persephin
GFRα: GFL receptor α
GDF15: Growth and differentiation factor 15
GFRAL: GFRα-like protein
Rac: Rat sarcoma virus (Ras)-related C3 botulinum toxin substrate
GPI: Glycosylphosphatidylinositol
PM: Plasma membrane
sGFRα: Soluble GFRα
pY: Phosphorylated tyrosine
SH2: Src homology 2
PTB: Phosphotyrosine-binding
SH2-Bβ: SH2 adaptor protein Bβ
PLCγ: Phospholipase Cγ
PKC: Protein kinase C
FRS2: Fibroblast growth factor receptor substrate 2
Shc: Src-homology collagen
IRS1/2: Insulin receptor substrate 1 and 2
DOK: Downstream of kinase
PI3K: Phosphatidylinositol-3 kinase
AKT: Protein kinase B
Ras: Rat sarcoma virus
MAPK: Mitogen-activated protein kinase
SHP-2: SH2 containing protein tyrosine phosphatase 2
GAB1/2: Grb2-associated binding protein 1 and 2
Myr: Myristoylation
NCAM: Neuronal cell adhesion molecule
TRK: Tropomyosin receptor kinase
FGFR: Fibroblast growth factor receptor
EGFR: Epidermal growth factor receptor
LOV: Light oxygen voltage sensing
PD: Parkinson’s disease
PHR: Photosensitive domain
CRY2: *Arabidopsis* Cryptochrome 2
SNc: Substantia nigra
PCR: Polymerase chain reaction
AAV: Adeno-associated virus
PEI: Polyethyleneimine
HEK: Human embryonic kidney
DMEM: Dulbecco’s modified Eagle’s medium
FBS: Fetal bovine serum
PEG: Polyethylene glycol
P/S: Penicillin–streptomycin
DIV: Days in vitro
NA: Numerical aperture
ROI: Region of interest
LED: Light-emitting diode
Avertin: 2,2,2-Tribromoethanol
PBS: Phosphate-buffered saline
PFA: Paraformaldehyde
NDS: Normal donkey serum
RT: Room temperature
pS6: Phosphorylated S6 ribosomal protein
TH: Tyrosine hydroxylase
DAPI: 4′,6-Diamidino-2-phenylindole
SDS-PAGE: Sodium dodecyl-sulfate polyacrylamide gel electrophoresis
TBS: Tris-buffered saline
TBST: TBS containing 0.1% Tween-20
pERK1/2: Phosphorylated p44/42 MAPK (ERK1/2)
GAPDH: Glyceraldehyde 3-phosphate dehydrogenase
T_1/2_: Activation half-life (time to reach the half-maximal response)
CI: Confidence interval
C/N: Cytosol-to-nucleus ratio
AKT-PH: Pleckstrin homology domain of AKT1
EKR-KTR: ERK kinase translocation reporter
PIP_2_: Phosphatidylinositol 4,5-bisphosphate
PIP_3_: Phosphatidylinositol 3,4,5-bisphosphate
Elk1: ETS Like-1 protein
Rho: Ras homologous
DAT: Dopamine transporter
DG: Dentate gyrus
IHC: Immunohistochemistry
NGF: Nerve growth factor
GEF: Guanine nucleotide exchange factor
ARP3: Actin-related protein 3
PAK: P21-Activated kinase
