# Supplementary material for: Optogenetic dissection of RET signaling reveals robust activation of ERK and enhanced filopodia-like protrusions of regenerating axons
Source: Mol Brain. 2023 Jul 4;16:56. doi: 10.1186/s13041-023-01046-6 (PMC10320947; doi:10.1186/s13041-023-01046-6)
Supplement: Supplementary file 1 — Additional file 1: Figure S1. Comparison of the structures of RET and its chimeric variants used for transduction of downstream signaling pathways. Figure S2. Robust clustering of Grb2 induced by activation of optoRET. Figure S3. Comparison of AKT and ERK signaling activation by GDNF, optoRET, and optoTrkB. Figure S4. Exponential curve fitting of AKT and ERK signaling activation. Figure S5. Comparison of the effect of dynamic stimulation of optoRET. Figure S6. Retrograde AKT and ERK signal transmission by optoRET. Figure S7. Flower-like F-actin structural reorganizations induced by the local photoactivation of optoRET. Figure S8. Inhibition of Cdc42 blocks the photoactivated F-actin structural reorganization. Figure S9. Axotomy of cultured neurons and optoRET activation with blue LED illumination. [file 13041_2023_1046_MOESM1_ESM.pdf]

Supplementary Information

Optogenetic dissection of RET signaling reveals robust activation of ERK and enhanced filopodia-like protrusions of regenerating axons

Bobae Hyeon[1], Heeyoung Lee[1], Nury Kim[2] and Won Do Heo[1,3]*

[1]Department of Life Sciences, Korea Advanced Institute of Science and Technology (KAIST), 291 Daehak-ro, Yuseong-gu, Daejeon, 305-701, Republic of Korea

[2]Center for Cognition and Sociality, Institute for Basic Science (IBS), Daejeon, Republic of Korea

[3]KAIST Institute for the BioCentury, Korea Advanced Institute of Science and Technology (KAIST), 291 Daehak-ro, Yuseong-gu, Daejeon, 305-701, Republic of Korea

*Corresponding author: wondo@kaist.ac.kr

Table of Contents

Supplementary Figure 1.	Comparison of the structures of RET and its chimeric variants used for transduction of downstream signaling pathways.
Supplementary Figure 2.	Robust clustering of Grb2 induced by activation of optoRET.
Supplementary Figure 3.	Comparison of AKT and ERK signaling activation by GDNF, optoRET, and optoTrkB.
Supplementary Figure 4.	Exponential curve fitting of AKT and ERK signaling activation.
Supplementary Figure 5.	Comparison of the effect of dynamic stimulation of optoRET.
Supplementary Figure 6.	Retrograde AKT and ERK signal transmission by optoRET.
Supplementary Figure 7.	Flower-like F-actin structural reorganizations induced by the local photoactivation of optoRET.
Supplementary Figure 8.	Inhibition of Cdc42 blocks the photoactivated F-actin structural reorganization.
Supplementary Figure 9.	Axotomy of cultured neurons and optoRET activation with blue LED illumination.
Supplementary Movie 1.	Photoactivated optoRET recruits its downstream signaling molecule, Grb2.
Supplementary Movie 2.	Optical enhancement of F-actin structural reorganization by optoRET.

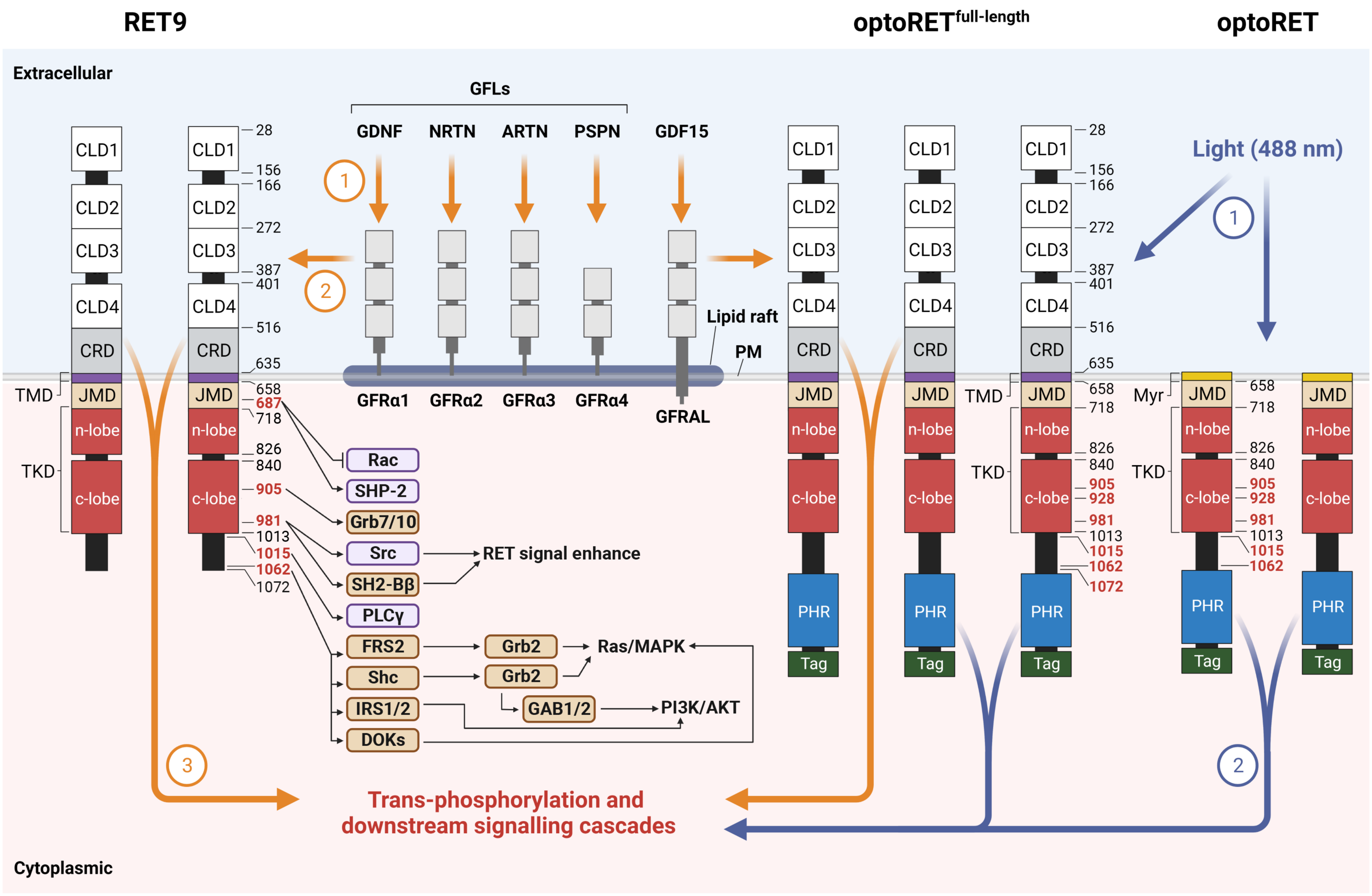

Figure S1. Comparison of the structures of RET and its chimeric variants used for transduction of downstream signaling pathways. The short-form of RET (RET9) comprises the extracellular cadherin-like domains (CLDs) 1–4 and cysteine-rich domain (CRD); transmembrane domain (TMD); and cytoplasmic region (CYR), including the juxtamembrane domain (JMD), kinase domain (TKD), and tail (aa 1013–1072). optoRET$^{full\text{-}length}$ comprises full-length RET9, the PHR and a tag (mCitrine or HA). optoRET comprises a myristoylation domain (Myr) anchored in the plasma membrane (PM), the cytoplasmic region of RET9 (aa 658–1062), the PHR and a tag (mCitrine or HA). The three steps involved in ligand-mediated activation of RET9 and optoRET$^{full\text{-}length}$ are indicated by orange arrows and the two steps involved in light-mediated activation of optoRET$^{full\text{-}length}$ and optoRET are indicated by blue arrows. The five RET ligands that bind to their preferred co-receptors are indicated in the first step of the ligand-mediated activation. The activated receptors can trans-phosphorylate tyrosine residues in the CYR, which in turn serve as docking sites for various adaptor and effector proteins. The key phosphorylated tyrosine residues are indicated in red, and directly docking adaptor or effector proteins are indicated with brown or purple boxes. The amino acid numbering follows that of human RET. Abbreviations: GDNF, glial cell line-derived neurotrophic factor; NRTN, neurturin; ARTN, artemin; PSPN, persephin; GDF15, growth and differentiation factor 15; GFLs, GDNF family ligands; GFRα1–4, GDNF family receptor α1–4; Rac, Rat sarcoma virus (Ras)-related C3 botulinum toxin substrate; SHP-2, protein tyrosine phosphatase-2; SH2-Bβ, Src homology 2 adaptor protein Bβ; PLCγ, phospholipase Cγ; FRS2, fibroblast growth factor receptor substrate 2; Shc, Src-homology collagen; IRS1/2, insulin receptor substrate 1 and 2; DOKs, downstream of kinase 4, 5 and 6; Grb7/10 and Grb2, growth factor receptor-bound protein 7, 10, and 2; GAB1/2, GRB2-associated binding protein 1 and 2; MAPK, mitogen-activated protein kinase; PI3K, phosphatidylinositol-3 kinase; and AKT, protein kinase B.

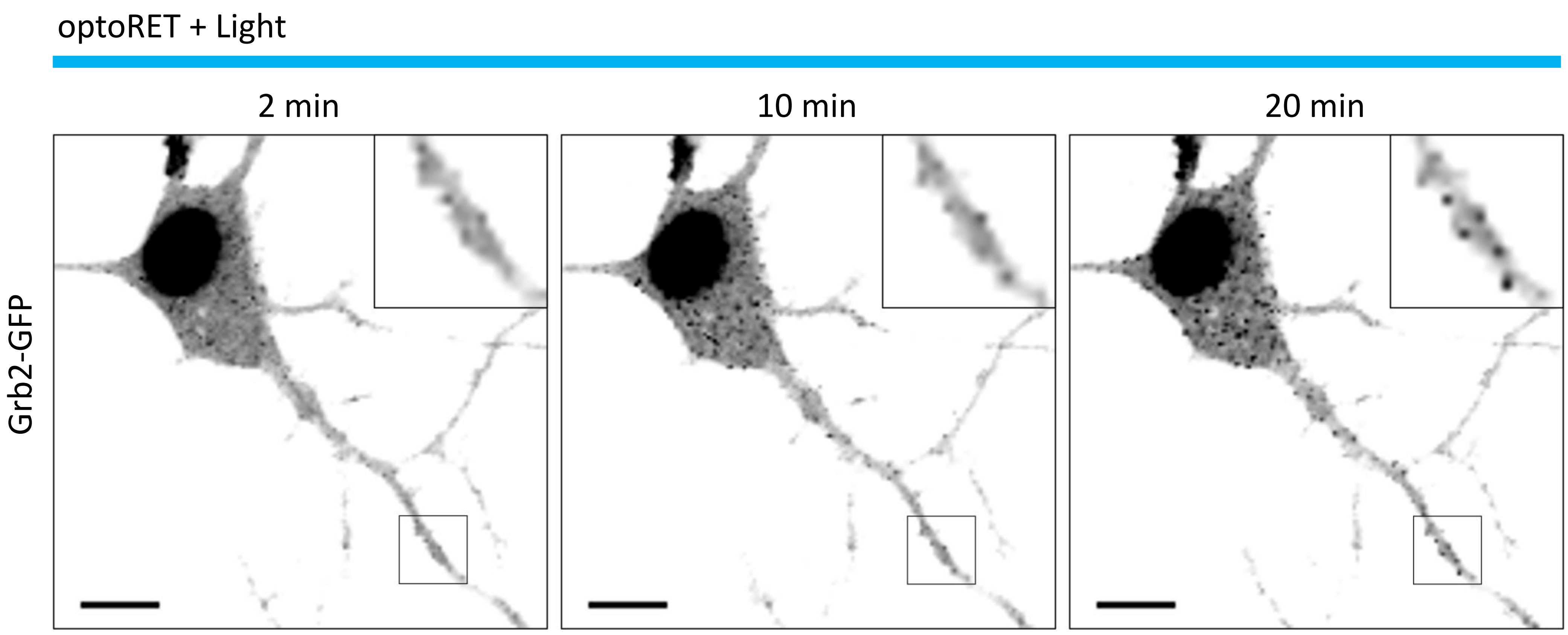

Figure S2. Robust clustering of Grb2 induced by activation of optoRET. Representative confocal images of neurons expressing optoRET(HA) and Grb2-GFP at 2, 10 and 20 min after co-imaging photoactivation. Scale bar = 10 µm.

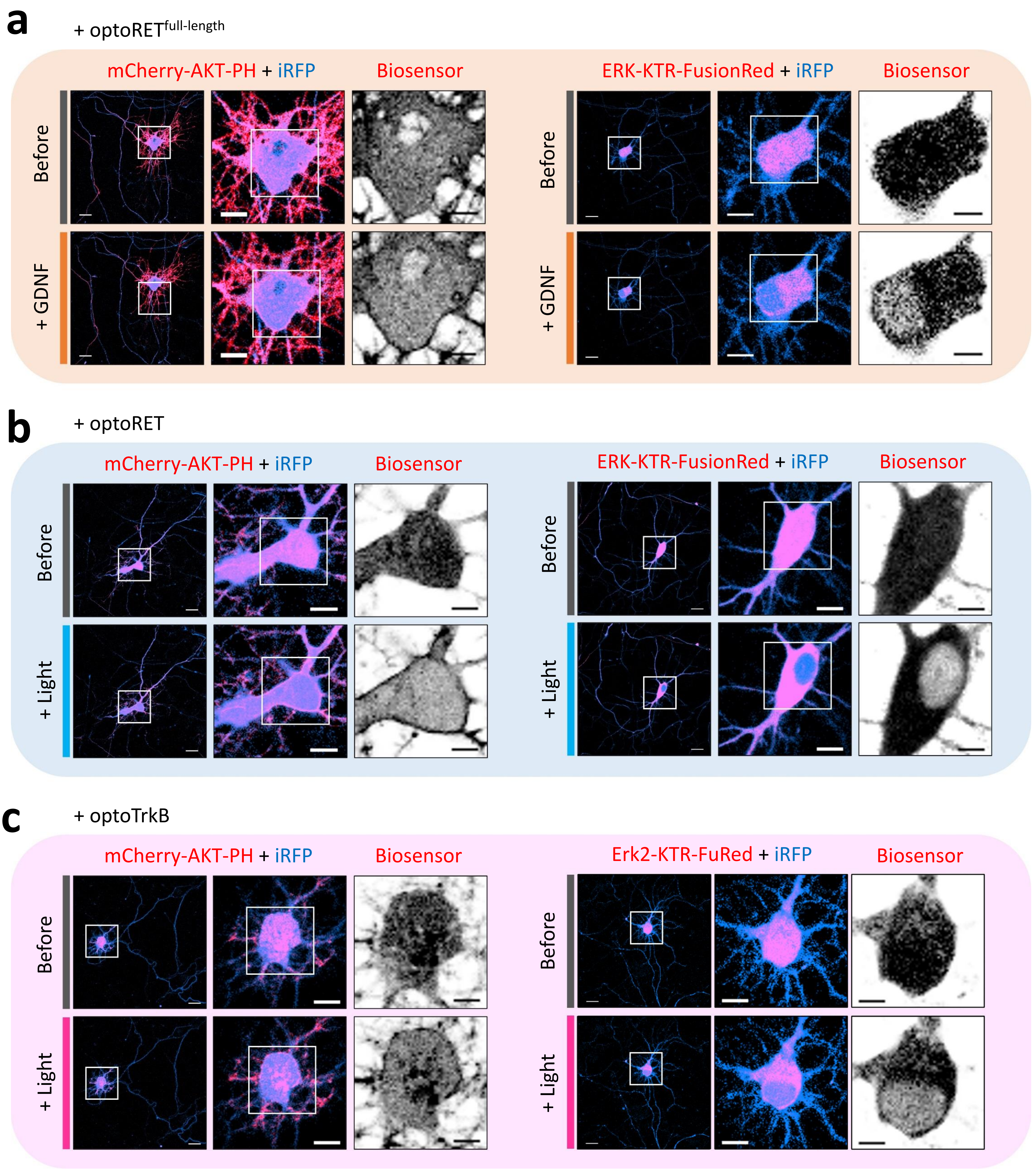

Figure S3. Comparison of AKT and ERK signaling activation by GDNF, optoRET, and optoTrkB. (**a**) Representative confocal images of cultured neurons expressing optoRET$^{full\text{-}length}$ together with iRFP and mCherry-AKT-PH (left) or ERK-KTR-FusionRed (right), before and after sustained stimulation with GDNF (50 ng/mL). (**b,c**) Representative confocal images of cultured neurons expressing optoRET (b) or optoTrkB (c) together with iRFP and mCherry-AKT-PH (left) or ERK-KTR-FusionRed (right), before and after sustained co-imaging photoactivation (~25 μW/mm^2). Scale bars = 20, 10 and 5 μm from the left to right images for each cell.

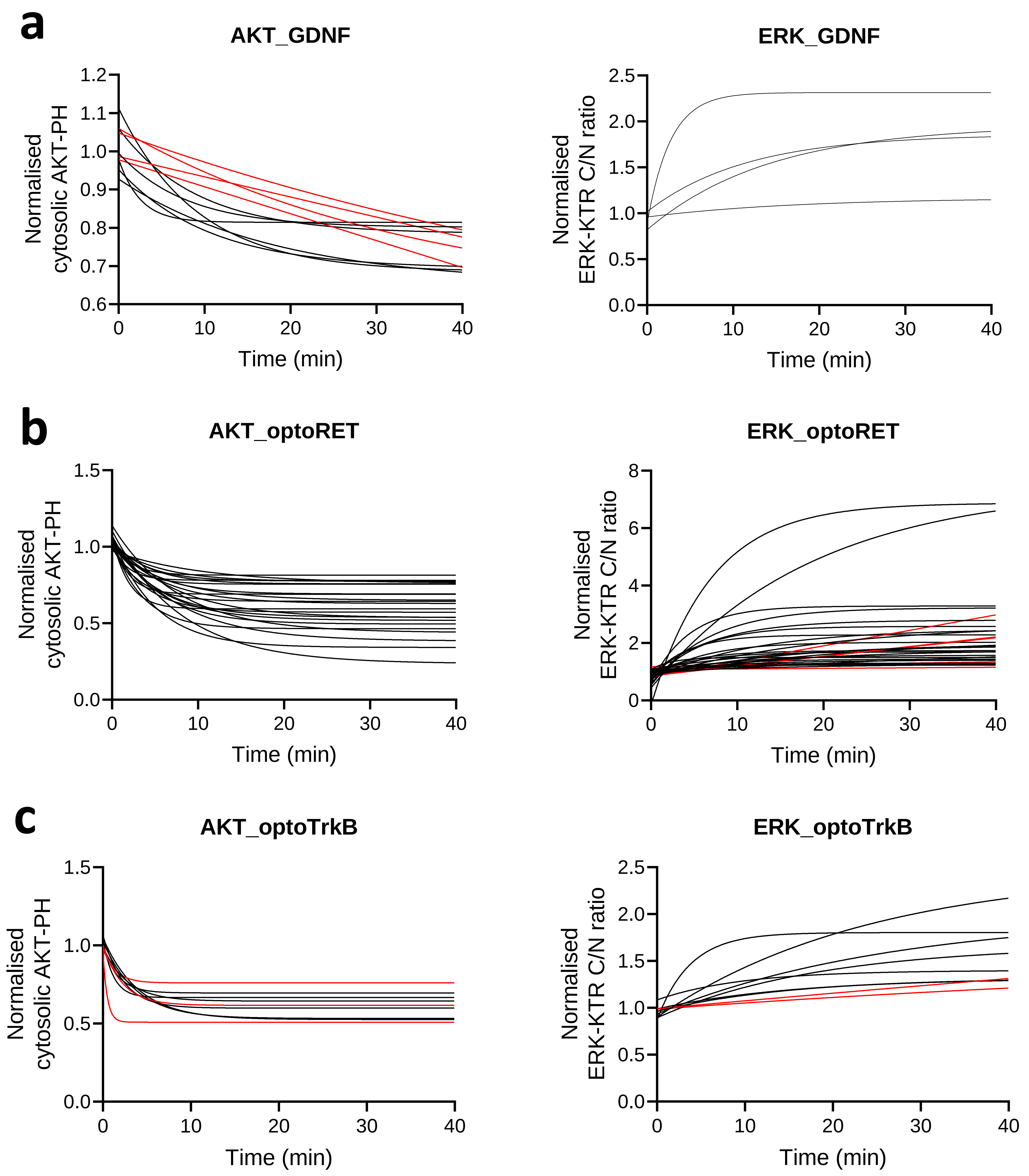

Figure S4. Exponential fitting curves of the AKT or ERK signalling activation. (**a-c**) The individual fitting curves of the individual cells with the successful AKT responses in the GDNF, optoRET and optoTrkB groups under the sustained stimulations. The fitted curves failed to have the 95% confident intervals (CI) are indicated in red. For the respective groups, n = total(failed fitting); AKT: n = 10(4), 23(0) and 9(3); ERK: n = 4(0), 30(6) and 9(2).

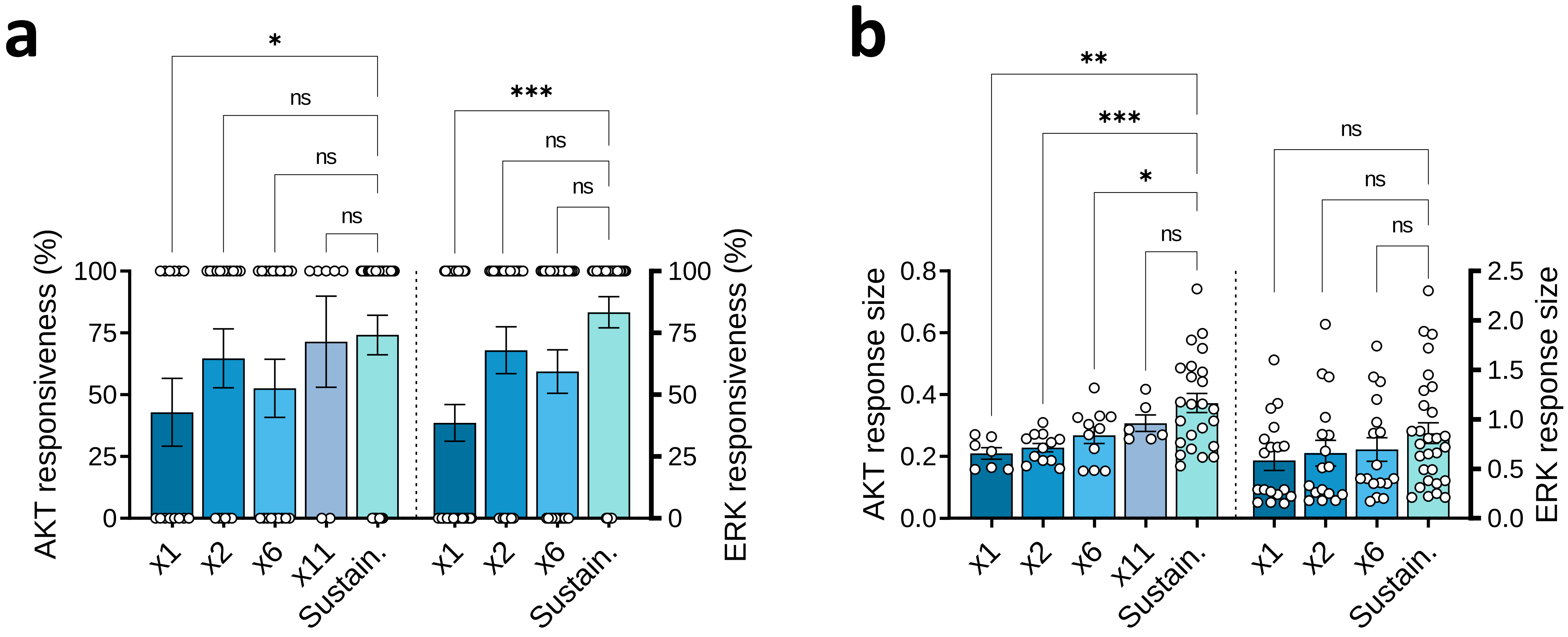

Figure S5. Comparison of the effect of dynamic stimulation of optoRET. (**a**) Comparison of responsiveness for AKT signaling to optoRET expressing groups with transient x1, x2, x6, x11 and sustained stimulation (n = 14, 17, 19, 7 and 31). For ERK signaling (right) with transient x1, x2, x6 and sustained groups (n = 44, 25, 32 and 36). (**b**) Comparison of response size for AKT (n = 7, 12, 11, 6 and 24) and for ERK (n = 18, 17, 17 and 29). Data are presented as means ± SEM (**p* < 0.05, **p < 0.01, ***p < 0.001; one-way ANOVA); ns, not significant (p > 0.05).

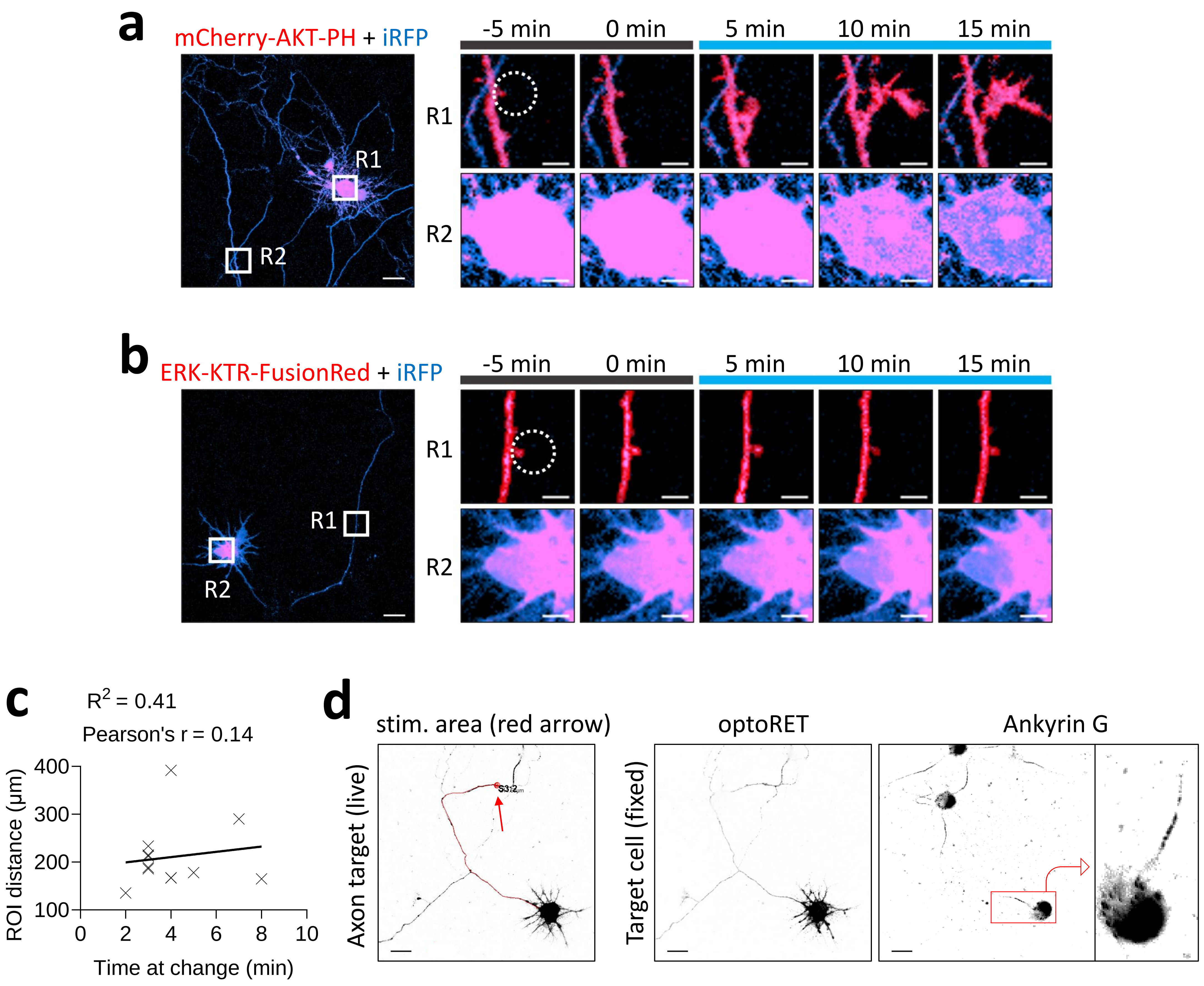

Figure S6. Retrograde AKT and ERK signal transmission by optoRET. (**a,b**) Representative time-lapse confocal images of the locally photoactivated neurons, expressing optoRET and mCherry-AKT-PH or ERK-KTR-FusionRed with iRFP, at the basal states (-5 and 0 min) and the photoactivated period (5, 10 and 15 min). The image on the left shows the positions of the target regions at basal state. The distal local regions (R1s), comprising the local photoactivation areas (dashed white circles), and the cell body regions (R2) are highlighted in the cropped images on the left. Scale bars = 20 and 10 μm. (**c**) Comparison of the distance between the soma to the stimulated area and the duration of a delayed period before the signal changes in the soma. The line indicate the fitting of total 12 points indicated by 'x' . (**d**) Example of a target axon, co-localized with the Ankyrin G at the axon initial segment. Scale bars = 20 μm.

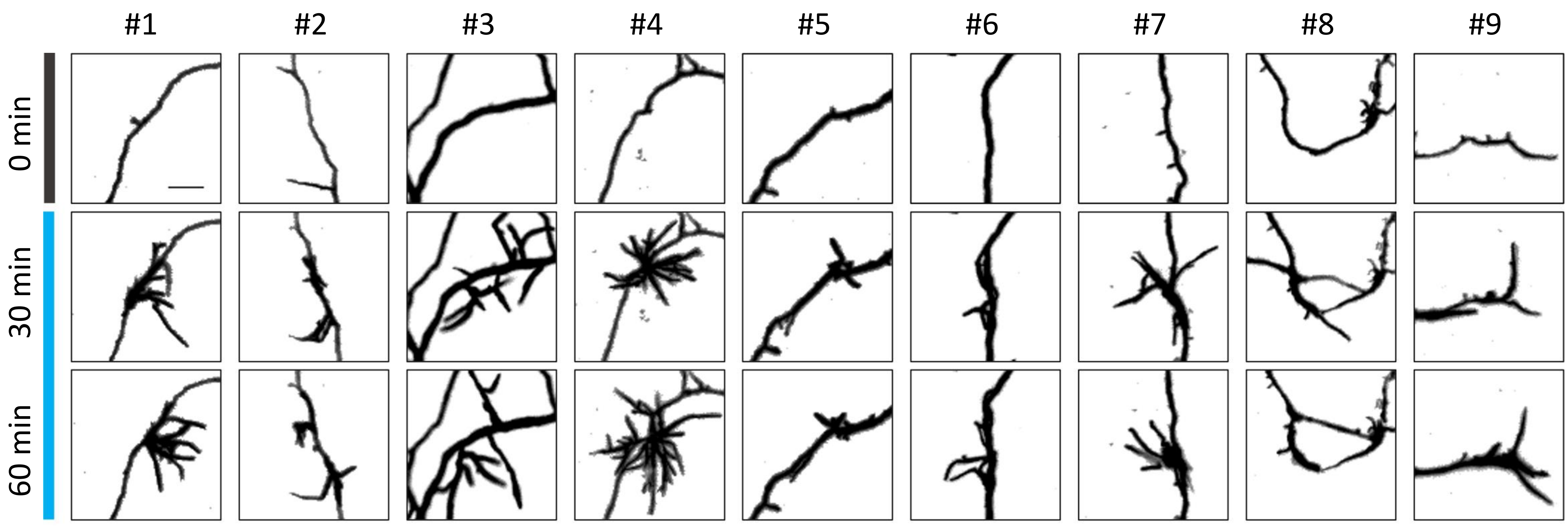

Figure S7. Flower-like F-actin structural reorganizations induced by the local photoactivation of optoRET. Representative time-lapse confocal images of the flower-like F-actin structural reorganization, visualized as the LifeAct signal, in response to local photoactivation of optoRET in ROIs (#1–9) of cultured neurons before (0 min) and after (30 and 60 min) photoactivation. Scale bar = 10 µm.

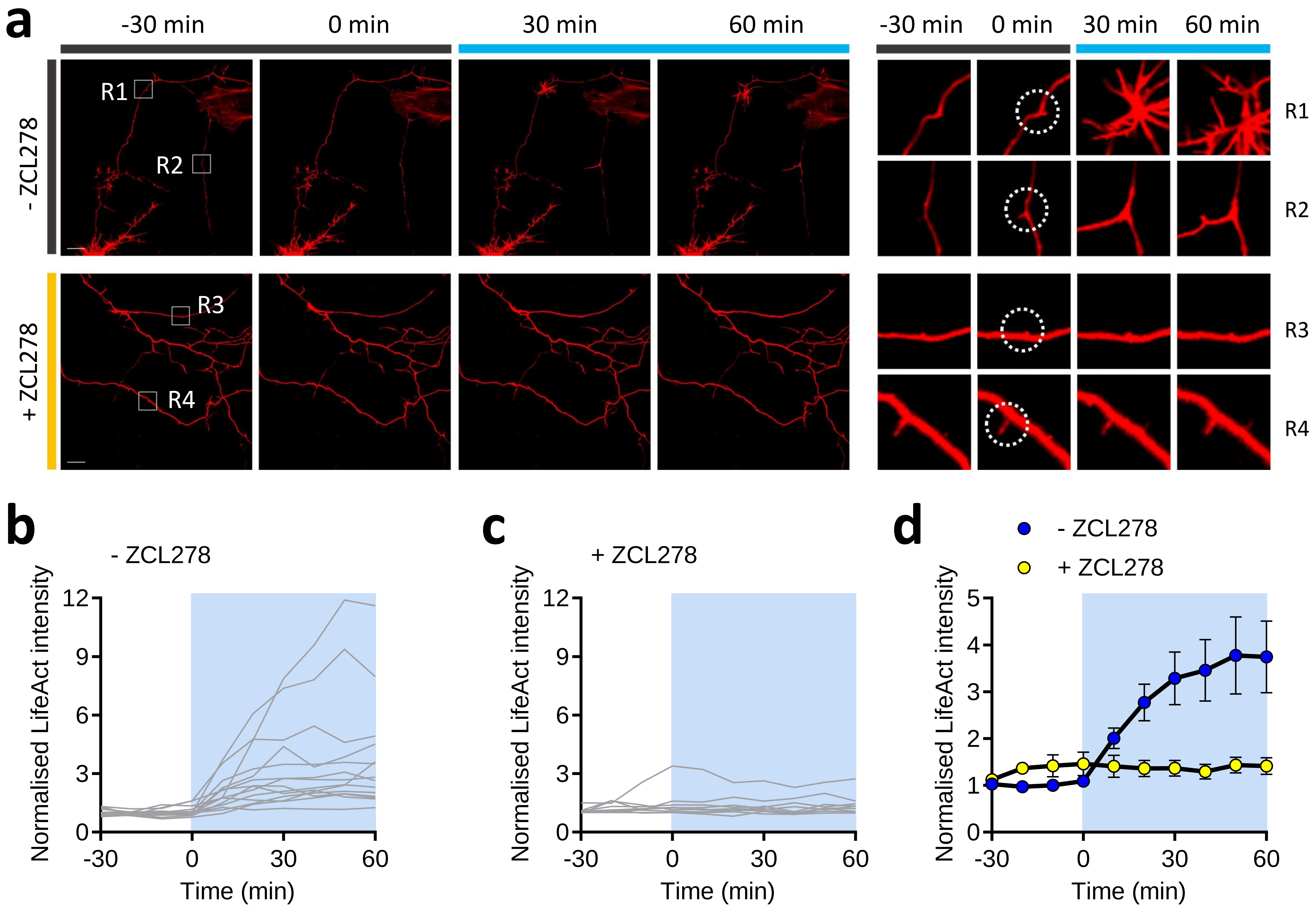

Figure S8. Inhibition of Cdc42 blocks the photoactivated F-actin structural reorganization. (**a**) Representative time-lapse confocal images of locally photoactivated neurons expressing optoRET and mCherry-LifeAct under basal conditions (-30 and 0 min) and during the photoactivated period (30 and 60 min) in the absence or presence of the Cdc42 inhibitor, ZCL278 (100 µM; 30 min pre-incubation). ROIs, corresponding to local photoactivation areas (dashed white circles), are shown in the cropped images on the right. Scale bars = 20 µm. (**b–d**) Quantification of normalized LifeAct intensities at ROIs in the two groups shown in panel (a). Data are presented as individual ROIs for each group (panels b and c) or as means ± SEM (panel d). Yellow, with ZCL278 (n = 14); blue, without ZCL278 (n = 9).

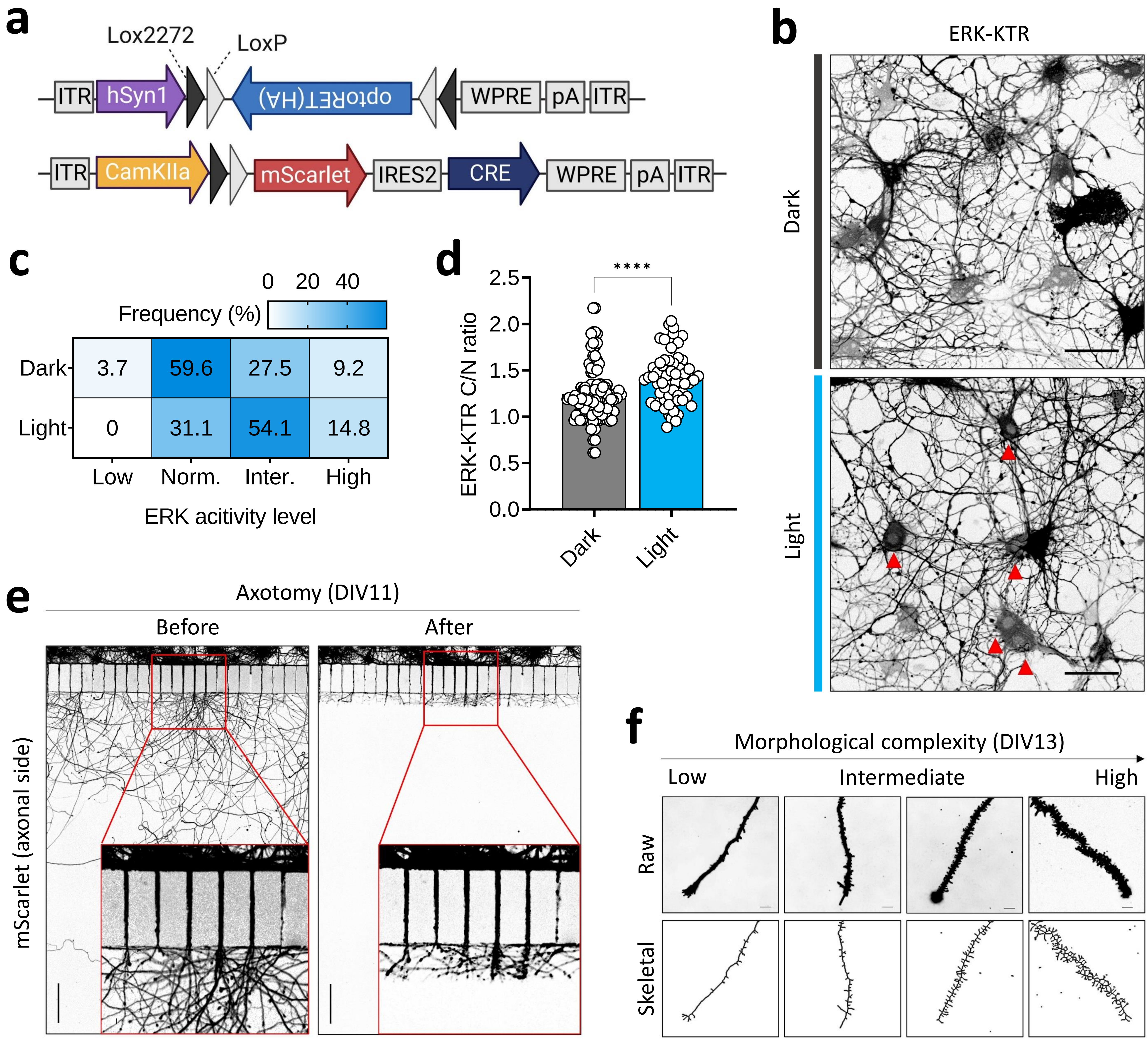

Figure S9. Axotomy of cultured neurons and optoRET activation with blue LED illumination. (**a**) Design of the viral vectors used to transduce optoRET into cultured neurons. For axotomy experiments, cultured neurons were transduced with AAV-DJ/8-CamKIIα-mScarlet-IRES2-CRE and AAV-DJ/8-DIO-hSyn1-optoRET for expression of mScarlet and optoRET. (**b**) Representative confocal images of cultured neurons co-transduced with AAV-DJ/8-CamKIIα-ERK-KTR-clover, with (Light group) or without (Dark group) illumination with a blue LED plate. Neurons with high ERK activity are indicated with red arrows. Scale bars = 50 µm. (**c**) Heatmap of the frequency distribution of neurons with low, normal, intermediate, or high levels of ERK activity. (**d**) Comparison of the EKR-KTR C/N ratio between Dark (n = 109) and Light (n = 61) groups. Data are presented as means ± SEM (*****p* < 0.0001; Kolmogorov-Smirnov test). (**e**) Representative confocal images of the axonal side of the microfluidics chip before and after axotomy at DIV11. Scale bars = 200 µm. (**f**) Representative raw and converted skeletal images of axon terminals from low to high axon morphological complexity. Scale bars = 10 µm.
