# Supplementary figures and images for: Optogenetic dissection of RET signaling reveals robust activation of ERK and enhanced filopodia-like protrusions of regenerating axons
Source: Mol Brain. 2023 Jul 4;16:56. doi: 10.1186/s13041-023-01046-6 (PMC10320947; doi:10.1186/s13041-023-01046-6)

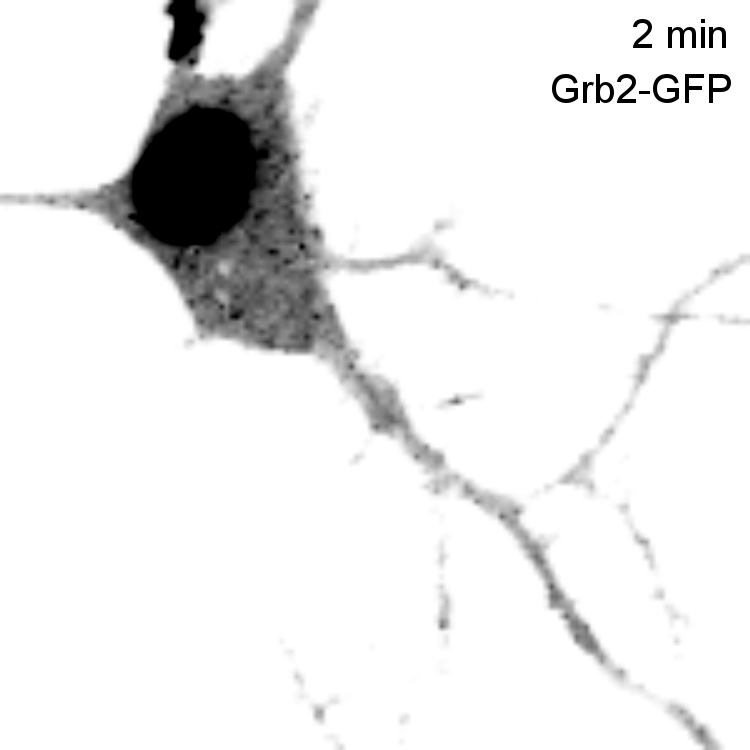

Supplement: Supplementary file 2 — Additional file 2: Movie S1. Photoactivated optoRET recruits its downstream signaling molecule, Grb2. [file 13041_2023_1046_MOESM2_ESM.gif]

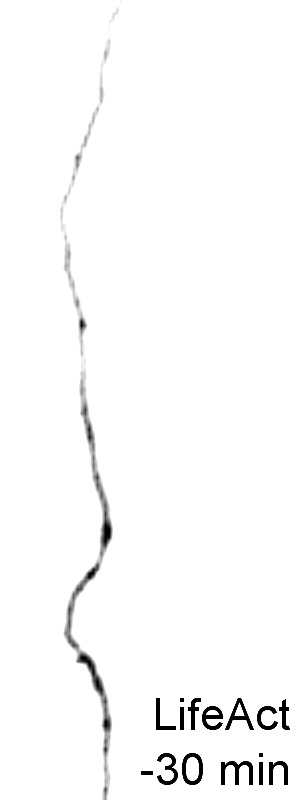

Supplement: Supplementary file 3 — Additional file 3: Movie S2. Optical enhancement of F-actin structural reorganization by optoRET. [file 13041_2023_1046_MOESM3_ESM.gif]
